# Synergistic Neurotoxicity of environmental Cadmium and Paraquat in Parkinsonism: Unveiling the Mito-ROS/OPA1/Caspase-3/GSDME-driven Apoptosis Axis

**DOI:** 10.7150/ijbs.126979

**Published:** 2026-02-18

**Authors:** Yufei Luo, Yingying Lu, Yancheng Tang, Xuejun Zhao, Junpu Wang, Yifan Liu, Sijia Jiang, Yuyuan Zhu, Jiaqi Qi, Linlin Xing, Chenghao Yan, Xu Liu, Cainian Huang, Haiji Wang, Haodong Xu, Liming Wang

**Affiliations:** 1 School of Biomedical Sciences, Hunan University, Changsha, China.; 2 Shenzhen Research Institute, Hunan University, Shenzhen, China.; 3 Department of Medical Research, Seventh Affiliated Hospital, Sun Yatsen University, Shenzhen, Guangdong, China.; 4 Department of Pathology, Xiangya Hospital, Central South University, Changsha, China.; 5 Department of Pathology, Xiangya School of Basic Medical Sciences, Central South University, Changsha, China.; 6 Department of Orthopaedics, The Second Xiangya Hospital of Central South University, Changsha, China.

**Keywords:** cadmium-paraquat co-exposure, mitochondrial apoptosis, Parkinsonian neurodegeneration, reactive oxygen species, OPA1 cleavage, caspase-3/GSDME axis

## Abstract

The increasing environmental presence of cadmium (Cd) and paraquat (PQ), driven by industrial emissions and overuse of herbicide, poses heightened risks for neurodegenerative disorders. Although each of these toxins can independently induce neuronal damage, the synergistic neurotoxic effects resulting from chronic, low-dose co-exposure to Cd and PQ remain inadequately understood. This study demonstrates that exposure to subtoxic levels of Cd and PQ concurrently induces neuronal cell death and contributes to Parkinson's disease (PD)-like symptoms. Mechanistically, chronic co-exposure to Cd and PQ triggers a marked overproduction of mitochondrial ROS (mito-ROS), which impairs OPA1 processing and results in mitochondrial fragmentation. This mitochondrial dysfunction subsequently triggers caspase-3 activation, leading to GSDME cleavage and its translocation to the mitochondria, ultimately promoting neuronal apoptosis. Furthermore, our* in vivo* studies demonstrate significant mitochondrial dysfunction and loss of nigrostriatal dopaminergic neurons, resulting in motor deficits and cognitive impairments in mice co-exposed to these toxins. Collectively, our findings reveal a novel molecular mechanism involving the mito-ROS/OPA1/caspase-3/GSDME pathway in environmentally-induced PD-like pathology, thereby offering potential therapeutic insights for PD treatment.

## Introduction

Parkinson's disease (PD) is the second most prevalent neurodegenerative disorder, clinically characterized by motor symptoms (e.g., bradykinesia, tremors), as well as non-motor symptoms (e.g., psychiatric disturbances, cognitive decline) 1. Pathologically, PD is marked by the selective loss of dopaminergic neurons in the substantia nigra (SN) pars compacta and the presence of intracellular Lewy bodies. Aging remains the primary risk factor for PD, with an incidence of 1-3% in individuals over 60 years old, increasing to approximately 5% in those over 85 2. Approximately 5-10% of PD cases are familial, with genome-wide association studies identifying key susceptibility genes including *SNCA, PINK1, PARK7, LRRK2,* and *PRKN* 1. While the majority of PD cases are sporadic, twin studies estimate a heritability of approximately 0.19 for these idiopathic cases, with common SNCA variants emerging as consistent risk factors 2. Importantly, this modest heritability highlights the substantial role of environmental factors in PD pathogenesis. Supporting this notion, epidemiological evidence has established significant associations between PD risk and environmental exposures, particularly to pesticides and heavy metals 3.

Paraquat (PQ) is currently the most widely used broad-spectrum, fast-acting contact herbicide in global agriculture 4. Despite being banned or restricted in many countries due to its acute toxicity (particularly irreversible lung injury), and lack of effective antidotes 5, 6, PQ remains extensively used in developing countries. This widespread use has led to substantial environmental contamination, with PQ ranking among the top five most detected pesticide residues in agricultural soils worldwide 7. Environmental monitoring reveals concerning PQ concentrations in water system, ranging from 0.3 to 13.7 μg/L in Barcelona or Spain's natural waters 8-exceeding the WHO's 10 μg/L safety threshold. Such contamination facilitates PQ's entry into ecosystems and food chains, raising significant public health concerns. Long-term exposure to PQ can cause toxic and teratogenic effects in humans and mammals 9. Notably, growing evidence links PQ exposure to PD risk, likely through the mechanisms involving oxidative stress and mitochondrial dysfunction. As a potent oxidant, PQ that significantly increases the production of free radicals 10 and disrupts redox homeostasis, as evidenced by reduced superoxide dismutase activity and elevated malondialdehyde levels in the hippocampus of mice 11. Importantly, PQ damages to mitochondrial proteins and enhances free radical generation, exacerbating the degeneration of dopaminergic neurons 12, 13 - a hallmark of PD pathology.

Cadmium (Cd), a toxic heavy metal, is widely used in industrial applications, including PVC stabilizers, battery production, and pigments 14. Widespread industrial emissions have resulted in significant environmental contamination, with the Cd content in groundwater exceeding the WHO safety threshold (3 μg/L) by 3-20 folds in multiple regions (Pakistan: 10 μg/L, India: 60 μg/L, and China: 39.59 μg/L 15-17. Of particular neurotoxicological concern, Cd can cross the blood-brain barrier, accumulating in neural tissues. Several epidemiologic studies have demonstrated that individuals with neurological disorders, including Alzheimer's disease and PD, exhibit significantly elevated blood or urinary Cd levels compared to healthy controls 18, 19. Furthermore, mechanistic studies have revealed that Cd exposure induces oxidative stress, inflammation, mitochondrial dysfunction, and apoptosis, all of which contribute to neuronal damage 20, 21. Notably, recent studies have demonstrated the amplified toxicity of Cd when co-exposure with polystyrene nanoplastics could synergistically cause ferroptosis/PANoptosis ultimately aggravating kidney/cardiomyocyte damage 22, 23. As mentioned above, both Cd and PQ share critical neurotoxic properties, specifically bioaccumulate through the food chain and tend to accumulate in the brain, where they induce oxidative stress and mitochondrial damage. However, the effects of chronic, low-dose co-exposure to Cd and PQ on neuronal viability and the development of PD remain largely unclear.

In this study, we demonstrated that co-exposure to subtoxic Cd and PQ synergistically induces neuronal apoptosis and recapitulates PD-like pathology. Mechanistically, combined exposure induces excessive mitochondrial reactive oxygen species (mito-ROS) production, leading to mitochondrial dysfunction predominantly via impaired OPA1 processing. This damage subsequently activates the caspase cascade and cleaves GSDME, which translocates to the mitochondria and ultimately drives apoptosis, degeneration of dopaminergic neurons, and the hallmark motor and cognitive deficits. Our findings identify the mito-ROS/OPA1/caspase-3/GSDME axis as a critical pathway bridging environmental toxin exposure to PD pathogenesis and provide new molecular targets for therapeutic intervention against PD.

## Materials and Methods

### Reagents

(CH_3_CO_2_)_2_Cd·xH_2_O (229490), paraquat (856177) and Mito-Tempo (SML0737) were purchased from Sigma (St. Louis, MO, United States). N-acetyl-L-cysteine (NAC) (HY-B0215), Q-VD (HY-12305), necroptosis inhibitor necrostatin-1 (Nec-1) (HY-15760), ferrostatin-1 (Fer-1) (HY-100579), necrostatin-1s (Nec-1s) (HY-14622) was supplied by MedChemExpress. CELLSAVING^TM^ (C40050) was purchased from NCM Biotech. Anti-GAPDH (60004-1, 1:5000), anti-β-actin (66009-1, 1:5000), anti-Tyrosine hydroxylase (TH) (25859-1, 1:1000) were obtained from Proteintech. Anti-caspase-3 (9662, 1:1000), anti-cleaved-caspase-3 (9661, 1:1000), anti-caspase-9 (9502S, 1:1000), anti-cleaved-PARP (5625T, 1:1000), anti-OMA1 (95473S, 1:1000), anti-OPA1 (80471S, 1:1000), anti-MFN1 (14739, 1:1000), anti-MFN2 (11925, 1:1000), anti-pS616 DRP1 (4494, 1:1000), anti-pS637 DRP1 (6319, 1:1000) and anti-GSDMD (39754S, 1:1000) were procured from Cell Signaling Technology. TOM20 (FL-145 and 17764, 1:200) was from Santa Cruz Biotechnology. Anti-PARP (T40050, 1:1000) was supplied by Abmart. Anti-GSDME (ab215191, 1:1000) was obtained from Abcam. BAX (610983, 1:1000) was from BD Biosciences. Cytochrome c (P20788, 1:200) was from ProMab Biotechnologies, Inc. Anti-BAX (6A7, NBP1-28566, 1:1000) was supplied by Novus Biologicals. Anti-VDAC (ab15895, 1:1000) was obtained from Abcam. HRP Goat Anti-Mouse IgG (H+L) (RS0001, 1:10000) and HRP Goat Anti-Rabbit IgG (H+L) (RS0002, 1:10000) were provided by Immunoway. The following secondary antibodies were purchased from Thermo Fisher Scientific: Alexa488 Anti-Mouse (A-32723, 1:1000), Alexa488 Anti-Rabbit (A-32731, 1:1000), Alexa555 Anti-Mouse (A-32727, 1:1000), Alexa555 Anti-Rabbit (A-32732, 1:1000) and Alexa647 Anti-Mouse (A-32728, 1:1000). Polyvinylidene fluoride (PVDF) membrane was supplied by Millipore (USA).

### Cell lines, cell culture and cell transfections

The human neuroblastoma cell line (SH-SY5Y) was obtained from the Stem Cell Bank, Chinese Academy of Sciences (Shanghai, China). SH-SY5Y cells were cultured in Dulbecco's Modified Eagle's Medium supplemented with 10% fetal bovine serum (FBS; Gibco) and 1% penicillin-streptomycin (P/S; GENVIEW, GA3502). Cells were maintained at 37 °C in a humidified incubator with 5% CO₂ and were routinely tested to confirm the absence of mycoplasma contamination.

To generate stable OPA1-FL (full-length) and OPA1-ΔS1 (S1-site cleavage-resistant) cell lines, we cloned full-length or S1-site mutations human OPA1 cDNA into a lentiviral vector. Lentiviruses were produced by transfecting HEK293T cells with these vectors and packaging plasmids (psPAX2/pMD2.G). Then SH-SY5Y were infected with concentrated viruses in the presence of polybrene, and selected with puromycin for 3 days to eliminate non-transduced cells. Stable expression was validated by Western blot analysis.

### Small interfering RNA (siRNA) transfection and lentivirus infection

Small interfering RNA (siRNAs) targeting *OMA1* were synthesized by Ribobio and transfected to SH-SY5Y cells with lipofectamine™ RNAiMAX (Invitrogen, 13778150) according to the manufacturer's instructions. Briefly, Lipofectamine™ RNAiMAX was first diluted in Opti-MEM™ (Gibco, 31985070) and incubated for 5 min at room temperature. Subsequently, the diluted siRNA was combined with the RNAiMAX solution and incubated for an additional 15 min at room temperature to facilitate complex formation. The mixture was then added dropwise to the cells in fresh complete medium. Cells were subsequently incubated at 37℃ with 5% CO₂ for 48 h. Target sequences of the siRNAs used in this study are as follows:

si*OMA1*: 5ʹ-ATGGATACTCTTCCTATTCAA-3ʹ;

si*GSDMD* #1: 5'-GUGUGUCAACCUGUCUAUCAA-3'; si*GSDMD* #2: 5'-CAGCACCUCAAUGAAUGUGUA-3';

The shRNA oligonucleotides were annealed and cloned into the pLKO.1 lentiviral vector. HEK293T cells were then transfected with the recombinant lentiviral constructs and packaging plasmids (psPAX2 and pMD2.G) using polyethyleneimine-mediated transfection. After 48 hours, the viral supernatant was harvested, filtered, and used to infect target cells. Knockdown efficiency was confirmed by Western blot analysis. The shRNA sequences targeting GSDME were as follows:

shRNA-GSDME: 5'-GCAGCAAGCAGCTGTITAT-3'.

### MTT assay for cell viability assessment

Cells were plated in 96-well plates (5 × 10³ cells/well) and allowed to adhere for 24 h. Following aspiration of culture medium, cells were exposed to specified concentrations of Cd and/or PQ for 36 h. Subsequently, 20 μL MTT solution (5 mg/mL; Beyotime, ST316) was added to each well followed by 4 h incubation at 37 °C. After carefully removing supernatants, formazan crystals were solubilized with 150 μL DMSO, and optical density was quantified at 570 nm using a microplate reader.

### CCK-8 assay for proliferation analysis

Cells were seeded identically as described for MTT assay. Post-treatment with Cd and/or PQ under identical experimental conditions, 10 μL CCK-8 reagent (Beyotime, C0039) was administered to each well. Following 4 h incubation, absorbance measurements were performed at 450 nm to determine cell proliferation rates.

### Combination index (CI) analysis

To evaluate whether Cd and PQ exert synergistic, additive, or antagonistic cytotoxic effects, the CI value was calculated based on cell viability data. SH-SY5Y cells were exposed to Cd or PQ alone, or to their combinations at fixed concentration ratios for 36 h. Cell viability was assessed using the CCK-8 assay. The x-axis represents the fractional effect (Fa), indicating the proportion of cells affected, while the y-axis shows the corresponding CI value 24. A horizontal dotted line at CI = 1.0 serves as the threshold defining additive effect. Data points with a CI greater than 1.0, indicating antagonism, are plotted in blue and are primarily distributed at lower fractional effects. Data points with a CI less than 1.0, indicating synergy, are shown in red and are predominantly clustered at higher Fa levels. Notably, the specific drug combination investigated in this study (5 µM Cd and 150 µM PQ) is highlighted as a green circle.

### Propidium iodide (PI) uptake and Annexin V/PI staining analysis

Following Cd and/or PQ treatment, the culture medium with dead cells was collected, and adherent cells were detached and harvested with EDTA-free trypsin, then centrifuged at 1000 rpm for 5 minutes at 4°C. Following PBS washes, cell pellets were resuspended in binding buffer and stained with Annexin V-FITC and/or PI for apoptosis or PI uptake analysis (FXP018-100, 4A Biotech). Finally, each sample data was collected with FACSCalibur flow cytometry (BD Biosciences), and analyzed using FlowJo software (v10; Tree Star).

### Western blotting

Total protein from cultured cells and mouse brain tissue was collected and lysed in lysis buffer with 1% protease inhibitor cocktail (Targetmol.USA, C0001) and and phosphatase inhibitors (Targetmol.USA, C0004). Mitochondrial and cytosolic proteins were extracted as previously described 25. Protein concentrations were determined using a BCA protein assay kit (Beyotime, P0009). Equal amounts of protein were then separated by SDS-PAGE and transferred to a PVDF membrane. After blocking with 5% BSA in TBST for 1 hour at room temperature, membranes were incubated overnight at 4°C with primary antibodies. Following washes, membranes were incubated with HRP-conjugated secondary antibodies for 1 hour at room temperature. Visualization was conducted using the ECL (Beijing Zoman Biotechnology Co., Ltd, ZD310) and UltraSignal ECL reagent (4A Biotech, 4AW011), and the relative optical density of each band was quantified with ImageJ software (National Institutes of Health).

### Confocal microscopy

Cells were seeded 1 day in advance on the clean cover glass (after high-pressure sterilization) in 6-well culture plates. Plant the cells on the cover glass in culture plates at the desired density, followed by incubating at 37 ℃ and 5% CO_2_, waiting for the cells to adhere to the wall. Following treatment with Cd (5 μM) and/or PQ (150 μM) for 36 h, cells were harvested, washed twice with 1× PBS, and fixed with 4% paraformaldehyde for 30 minutes at room temperature. They were then permeabilized with 0.25% Triton X-100 for 15 minutes at room temperature. After blocking with 10% BSA for 1 h at 37°C, cells were incubated overnight at 4 °C with primary antibodies. Following PBS washes, cells were incubated with Alexa Fluor 488- or 594-conjugated secondary antibodies (1:1000; Thermo Fisher Scientific) for 1 h at room temperature. Images were acquired using a Zeiss LSM-980 confocal laser scanning microscope. The co-localized areas of cytochrome c and TOM20 were quantified using ImageJ by dividing the co-localized area by the total TOM20-positive area, and the fluorescence intensity of BAX6a7 was quantified using ImageJ.

### DNA ladder assay

Following 36 h of Cd (5 μM) and/or PQ (150 μM) treatment, cells were trypsinized and collected. After PBS washes, the genomic DNA was extracted and purified using a commercial DNA extraction kit (Beyotime, C0007) according to the manufacturer's instructions. Equal amounts of purified DNA were subjected to electrophoresis on a 1.0% agarose gel at 120 V for 30 minutes at room temperature. The DNA bands were visualized and photographed by Bio-Rad Chemidoc MP.

### Estimation of mito-ROS production

Cells were cultured in a 6-well plate, pretreated with 1 mM NAC or 100 μM Mito-Tempo for 1 h, followed by 36 h exposure to 5 μM Cd and/or 150 μM PQ. After PBS washes, cells were trypsinized and collected for staining with 1 μM MitoSOX Red (Thermo Fisher Scientific, M36008) for mito-ROS detection (37◦C for 30 minutes in the dark). Finally, each sample data was collected with FACSCalibur flow cytometry (BD Biosciences), and analyzed using FlowJo software (v10; Tree Star).

### Animals

C57BL/6 male mice (23 ± 2 g; Vital River Laboratory, Beijing, China) were acclimated for 1 week under standard conditions (24 ± 2 °C, 12-hour light/dark cycle, ad libitum access to food and water). Specifically, mice were randomly divided into 4 groups (n = 11/group): Control group (Received deionized water and intraperitoneal (i.p.) injections of 0.9% saline (0.2 mL, twice weekly), Cd group (Administered 25 mg/L cadmium acetate (CH_3_CO_2_)_2_Cd·xH_2_O via drinking water), PQ group (Treated with 5 mg/kg PQ which dissolved in 0.9% saline, i.p., twice weekly ), and Cd + PQ group (Received combined Cd water and PQ injections).Treatments lasted 8 weeks, and body weight monitored weekly. All animal experiments conducted in this study were performed in accordance with the guidelines of the Ethics Committee of Shenzhen Lingfu TopBiotech (Approval No.: TOPGM-IACUC-2024-0155).

The exposure dose of Cd used in this study was selected based on the lowest observed adverse effect level for long-term exposure in mice and environmental equivalent Cd exposure concentrations 26. While PQ dosage was optimized from previous studies demonstrating no observable nervous system damage 27. The PQ concentration used in this study was determined based on reported human exposure levels. Environmental monitoring data indicates airborne PQ concentrations of approximately 40 μg/m³, with estimated cumulative brain concentrations of 257-675 μM in individuals over 50 years old 28. Considering a typical daily respiratory volume (10-20 m³/day) and an average body weight of 50 kg, the corresponding daily human exposure is estimated at 0.01-0.02 mg/kg 29. Following interspecies extrapolation, this translates to an equivalent animal dosage of 1-2 mg/kg/day, which was adopted in our experimental design. After the final injection, motor behavior assessments were conducted by researchers blinded to group allocation. Mice were then euthanized for tissue collection. For molecular analyses, mice underwent cardiac perfusion with 0.9% saline, followed by rapid dissection of the bilateral striatum (STR) and SN. Tissues were rinsed with cold saline, snap-frozen in liquid nitrogen, and stored at -80°C. Additionally, brains were fixed in 4% paraformaldehyde for immunofluorescence staining with frozen section embedding agent (Solarbio LIFE SCIENCES, I7830) or 2.5% glutaraldehyde for transmission electron microscopy analysis. All procedures were performed by researchers blinded to treatment groups to avoid bias.

### Behavioral testing

All behavioral tests were carried out in the neurobehavioral room between 9:00 and 17:00, under established optimal conditions. All behavioral experiments were performed by experimenters who remained blinded to the group allocation during both data collection and data analysis.

### Open field test

The open field test was used to evaluate automatic motor behavior in mice. The setup consisted of a 50 cm × 50 cm square arena with 40 cm-high walls. Mice were placed in the center, and their movements were recorded for 5 minutes using a camera. Before testing, mice were acclimated to the environment for 3 days. After each session, the arena was cleaned with 75% alcohol to prevent contamination. The test was conducted under consistent lighting.

### Grasping test

A 1 mm diameter horizontal stainless-steel wire was suspended 30 cm above the ground, and mice were placed to grip the wire with their forepaws to assess their grasping ability for 10 seconds. Mice were scored based on whether they gripped with both hind legs (3 points), one hind leg (2 points), neither hind leg (1 point), or if they fell (0 points). Each mouse underwent three trials, and the average score was calculated.

### Rotarod experiment

The rotarod test was performed to evaluate motor coordination and balance in mice using a rotarod apparatus (Sansbio, Nanjing, China). Mice were placed on a rotating rod starting at 4 rpm, with the speed gradually increasing to 40 rpm over 5 minutes. Prior to testing, mice were trained for 3 days. The latency to fall (in seconds) was automatically recorded by the apparatus. Each mouse was tested three times with 5-minute intervals, and the average fall latency was calculated for statistical analysis.

### Pole climbing test

Placing mice head-upward on a cotton-wrapped wooden pole (1 cm in diameter, 55 cm high). A turn was defined as the point at which the mouse fully reoriented its head downward, indicating the initiation of descent. The time taken for mice to turn completely and descend the pole was recorded. If a mouse stopped midway or climbed in the wrong direction, the test was repeated. Mice were acclimatized for 3 days before testing. Each mouse was tested three times, with the average time recorded. The maximum duration limit is 60 s for each test.

### Morris water maze (MWM) test

MWM apparatus was used, featuring a circular water tank (100 cm diameter, 40 cm height) filled with opaque water at 23 ± 1 °C and 20 cm deep. An escape platform was hidden 2 cm under the water in one quadrant. The pool was surrounded by distinct distal spatial cues (e.g., geometric shapes and high-contrast visual markers) that remained fixed throughout both the training and probe trials. Mice underwent 5 days of training (4 trials/day, 60 s per trial). A trial ended when the mouse found the platform or after 60 s. Put the mice on the platform for 15 s if they could not find the platform within 60 s. On Day 6, during the probe test, the platform was removed and the mice had 90 s to navigate the pool. All data recorded via SMART software (Panlab, MA, USA).

### Tail suspension test

Mice were allowed to adapt to the test room for at least 2 h prior to testing. In brief, mice were securely fastened with an adhesive tape and hung head-down 20-25 cm above the floor. The immobility time was recorded over a 5-minute period using SMART software (Panlab, MA, USA).

### Statistical analysis

Data analysis was conducted using GraphPad Prism 8 (version 8.0.2). The selection of statistical tests was based on the results of the normality test. Results are presented as mean ± standard deviation (SD) from at least three independent experiments. One-way ANOVA followed by Bonferroni post hoc analysis was performed, and *p* < 0.05 was considered statistically significant.

## Results

### Co-exposure to subtoxic Cd and PQ induces synergistic cytotoxicity

**Figure 1:**
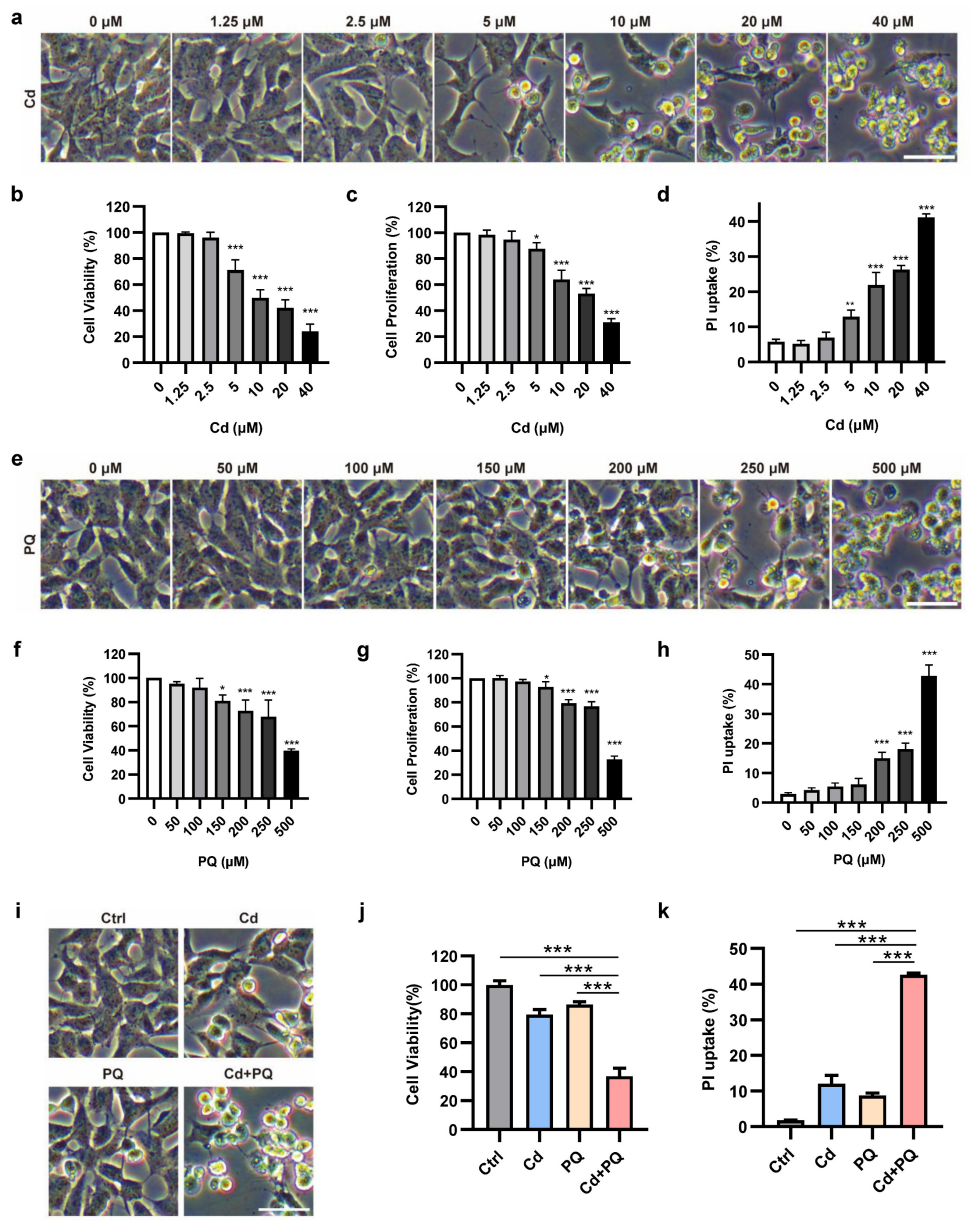
** Co-exposure to subtoxic Cd and PQ induces synergistic cytotoxicity. (a)** Morphological alterations in SH-SY5Y cells following 36 h of Cd exposure. **(b-d)** Assessment of cell viability **(b)**, cell proliferation **(c)**, and PI uptake **(d)** in SH-SY5Y cells treated with Cd for 36 h. **(e)** Microscopic analysis of the morphological characteristics of SH-SY5Y cells after 36 h of PQ treatment. **(f-h)** Measurements of cell viability **(f)**, cell proliferation** (g)**, and PI uptake **(h)** in SH-SY5Y cells treated with PQ for 36 h. **(i-k)** SH-SY5Y cells were treated with 5 μM Cd and/or 150 μM PQ for 36 h, showing dead cell morphology **(i)**, cell viability **(j)**, and PI uptake **(k)**. Scale bar, 100 μm. Data are presented as mean ± SD from three independent experiments. (*p < 0.05, ** p < 0.01, *** p < 0.001).

Previous studies have consistently demonstrated that Cd exhibits neurotoxic effects in various models in a dose-dependent manner 30, 31. To further investigate the neurotoxic effects of Cd, SH-SY5Y cells were exposed to a broad spectrum of concentrations (1.25, 2.5, 5, 10, 20, and 40 μM) (Fig. 1a). At concentrations exceeding 10 μM, Cd induced progressive morphological deterioration, characterized by cell rounding and detachment (Fig. 1a). Both MTT (Fig. 1b) and CCK8 (Fig. 1c) assays demonstrated that Cd significantly inhibited cell viability and proliferation in a dose-dependent manner, with pronounced cytotoxicity observed at concentrations above 10 μM. To further validate these findings, the PI uptake assay was conducted, revealing a substantial increase in PI uptake starting from 5 μM, indicative of cell membrane damage (Fig. 1d and Supplementary Fig. 1a). Based on these results, 5 μM Cd was established as the subtoxic concentration for subsequent co-exposure experiments.

To evaluate the cytotoxicity of PQ, MTT, CCK8, and PI uptake assays were conducted. At all measured endpoints, PQ concentrations below 150 µM failed to induce any observable morphological alterations or significant loss of cell viability (Fig. 1e-h and Supplementary Fig. 1b). Based on these findings, a concentration of 150 µM was established as the subtoxic dose for PQ in subsequent co-exposure experiments.

We subsequently assessed the cytotoxicity of subtoxic doses of Cd and PQ under co-exposure conditions. Drug interactions were analyzed using the CI method over a concentration series following 36 h of combined treatment. A CI value of less than 1.0 indicates synergy. Notably, the combination of 5 µM Cd and 150 µM PQ for 36 h showed strong synergy (CI < 1), highlighted by a green circle in Supplementary Fig. 1c. Consistent with these results, morphological observation (Fig. 1i) and functional assays—including MTT (Fig. 1j), CCK-8 (Supplementary Fig. 1d), and PI uptake (Fig. 1k and Supplementary Fig. 1e) all revealed significantly enhanced cytotoxicity in the co-exposure group compared to either agent alone. These findings indicate that co-exposure to subtoxic doses of Cd and PQ synergistically exacerbates neuronal damage, likely through cooperative toxicological mechanisms.

### Co-exposure to Cd and PQ induces apoptosis in SH-SY5Y cells

**Figure 2:**
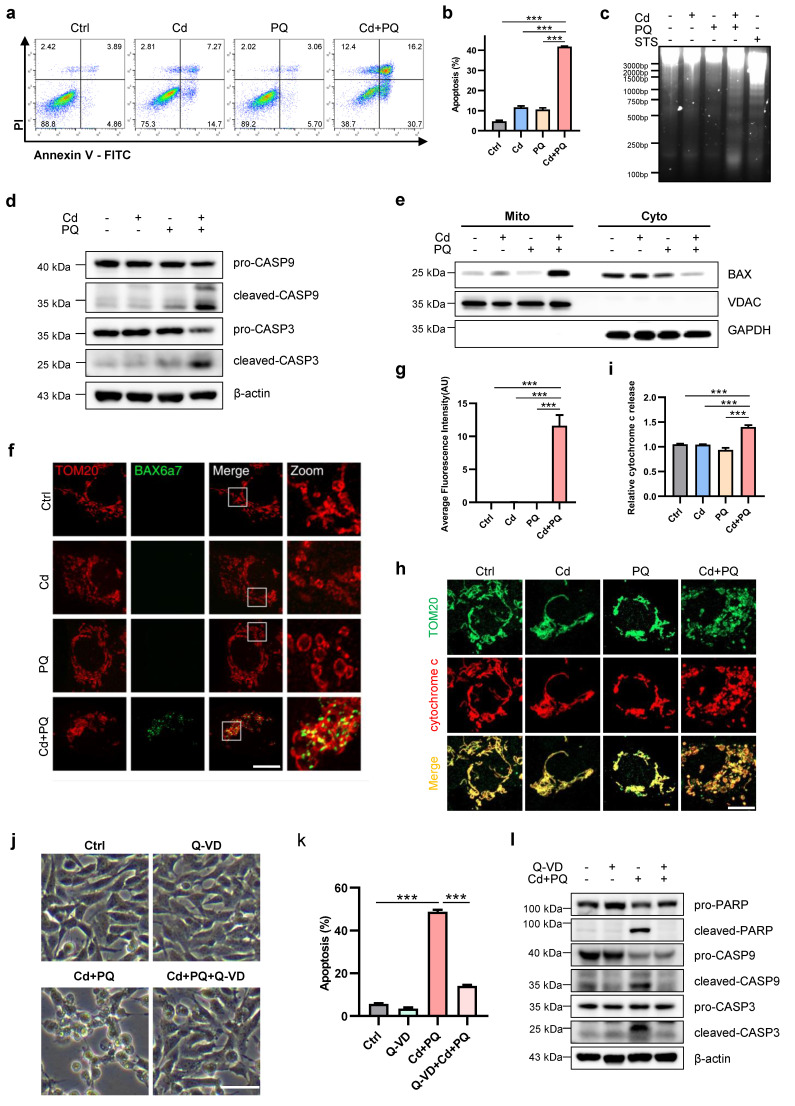
** Co-exposure to Cd and PQ induces apoptosis in SH-SY5Y cells.** SH-SY5Y cells were treated with 5 μM Cd and/or 150 μM PQ for 36 h, with or without a pre-treatment of 20 μM Q-VD for 1 h, to assess apoptosis levels. **(a)** Flow cytometry analysis was conducted using Annexin V-FITC/PI staining, and **(b)** quantitative data for apoptosis rates (%) were obtained. **(c)** Electrophoresis was performed to analyze the apoptotic DNA ladder extracted from SH-SY5Y cells exposed to Cd and/or PQ. Staurosporine (STS), a broad-spectrum kinase inhibitor, was used as a positive control for apoptosis induction. **(d)** The expression of caspase-9 (CASP9) and caspase-3 (CASP3) in SH-SY5Y cells was evaluated through Western blotting after treatment with Cd and/or PQ. **(e)** The mitochondrial (Mito) and cytosolic (Cyto) fractions were isolated and analyzed via Western blot to detect the translocation of BAX to the mitochondria. GAPDH and VDAC served as internal controls for the cytosolic and mitochondrial fractions, respectively.** (f)** Representative confocal microscopy images showing immunofluorescence staining for mitochondrial outer membrane marker TOM20 (red) and active BAX (green, detected with clone 6A7 antibody) in cells treated with Cd and/or PQ for 36 h. Scale bar: 20 μm. **(g)** Quantification of mitochondrial BAX activation in** (f)**. **(h)** Immunostaining for cytochrome c (red) and mitochondria (TOM20, green) in cells treated with Cd and/or PQ (scale bar: 20 μm). The relative cytochrome c release was quantified and presented in** (i)**. **(j)** Morphological characteristics of SH-SY5Y cells treated with Q-VD (20 μM, pre-treated for 1 h), Cd (5 μM) and PQ (150 μM), or both under the microscope. Scale bar, 100 μm. **(k)** Effect of Q-VD, Cd and PQ, or both intervention on apoptosis rate in SH-SY5Y cells.** (l)** Cells were treated with vehicle, Cd (5 μM) and PQ (150 μM), and Cd plus PQ and Q-VD (20 μM) for 36 h, followed by Western blot analysis to assess the expression of PARP, caspase-9 (CASP9) and caspase-3 (CASP3), β-actin was used as an internal control. Data are presented as mean ± SD from three independent experiments. (* *p* < 0.05, ** p < 0.01, *** p < 0.001).

To elucidate the specific cell death mechanism underlying Cd and PQ co-exposure-induced cytotoxicity, we first investigated apoptotic hallmarks in SH-SY5Y cells. Flow cytometric analysis of Annexin-V-FITC/PI staining revealed a significant increase in apoptosis rates in the co-exposure group compared to single treatments and the control (Fig. 2a, b). We further confirmed the occurrence of apoptosis using a DNA ladder assay. Notably, characteristic oligonucleosomal DNA fragmentation was observed exclusively following co-exposure to Cd and PQ. This pattern was consistent with the positive control (STS treatment) (Fig. 2c). Moreover, Western blot analysis showed elevated levels of apoptosis-related proteins, cleaved caspase-9 and cleaved caspase-3, in co-exposed cells compared to single treatments and the control (Fig. 2d). A critical event in intrinsic apoptosis is the release of cytochrome c from mitochondria into the cytosol, facilitated by pore-forming proteins such as BAX and BAK 32. Consistent with this, co-exposure significantly promoted the activation and mitochondrial translocation of BAX (Fig. 2e-g) and subsequent cytochrome c release from mitochondria into the cytosol (Fig. 2h-i).

To further validate that apoptosis, rather than other forms of cell death, occurs in co-exposed cells, pharmacological inhibitors targeting distinct cell death pathways were employed. Among these, only the apoptosis inhibitor Q-VD exhibited significant protective effects against cell death induced by co-exposure (Fig. 2j). In contrast, the ferroptosis inhibitor Fer-1, necroptosis inhibitor Nec-1 and Nec-1s displayed minimal protective effects (Supplementary Fig. 2a-f). Consistent with this observation, flow cytometry results revealed that Q-VD markedly attenuated apoptosis in cells exposed to Cd and PQ co-treatment (Fig. 2k). Additionally, Western blot analysis confirmed that the expression levels of cleaved PARP, cleaved caspase-9, and cleaved caspase-3 were substantially reduced in the Cd+PQ+Q-VD group compared to the co-exposure group (Fig. 2l). Collectively, these findings provide robust evidence that the cytotoxicity induced by Cd and PQ co-exposure is primarily mediated via caspase-dependent apoptosis.

### GSDME mediates Cd and PQ co-exposure-induced apoptosis

**Figure 3:**
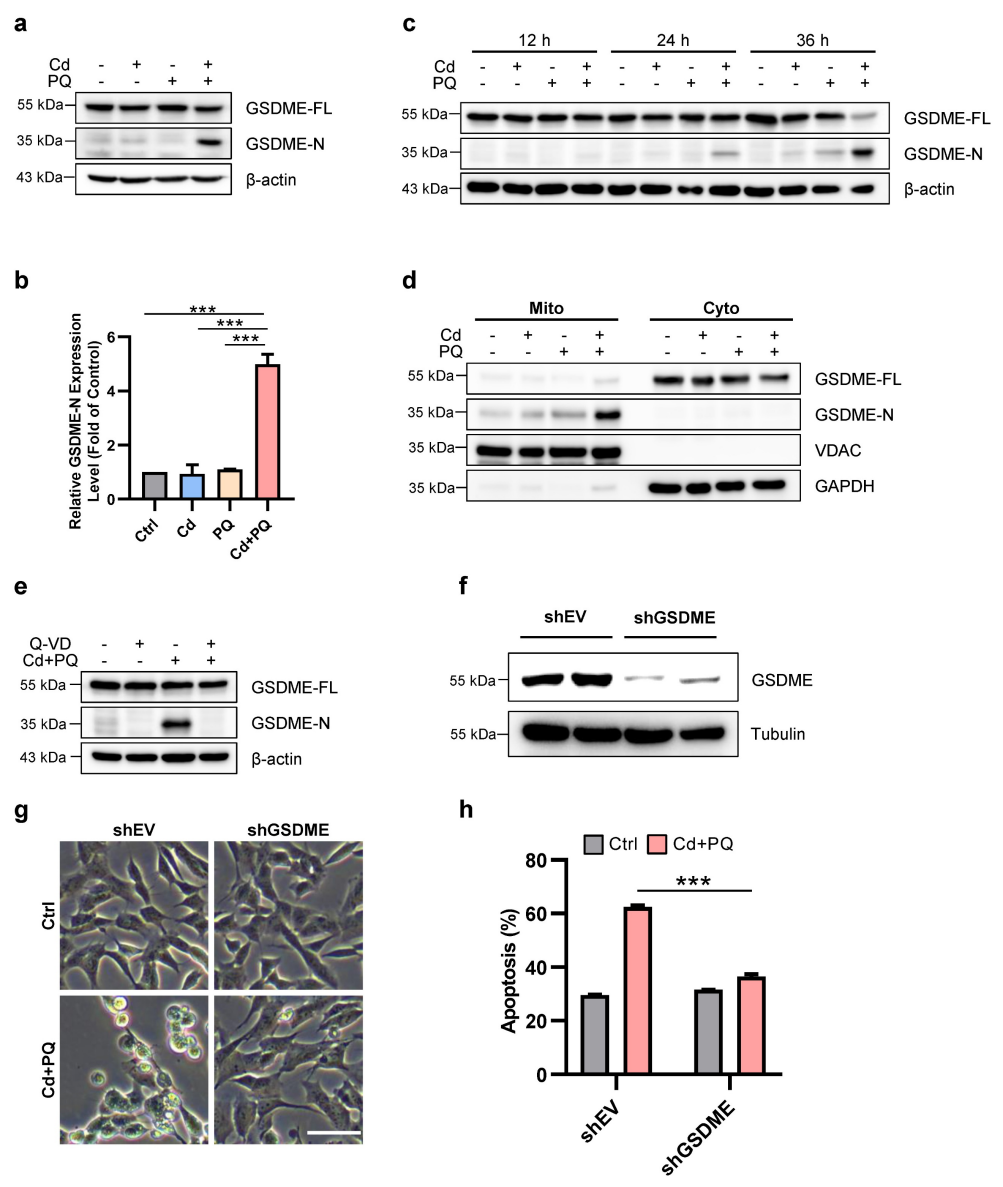
** GSDME mediates Cd and PQ co-exposure-induced apoptosis.** 5 μM Cd and/or 150 μM PQ were exposed to SH-SY5Y cells, and the following parameters were measured.** (a, b)** Western blot analysis of GSDME expression after 36 h. **(c)** The expression of GSDME in SH-SY5Y cells was tested by Western blot at different time points following treatment. **(d)** Representative Western blot showing the effects of 36 h co-exposure to Cd (5 μM) and PQ (150 μM) on GSDME protein expression in mitochondria (Mito) and cytoplasm (Cyto) of SH-SY5Y cells. **(e)** SH-SY5Y cells were pretreated with Q-VD (20μM) for 1 h before treatment with Cd (5 μM) and PQ (150 μM) for 36 h. Western blot was used to assess the cleavage of GSDME. **(f-h)** SH-SY5Y control (shEV) and GSDME knockdown (shGSDME) cells were generated. The efficiency of the GSDME knockdown was confirmed by Western blot **(f)**. The morphology of SH-SY5Y cells** (g)** and the percentage of apoptotic cells **(h)** were examined after 36 h of Cd (5 μM) and PQ (150 μM) treatment. Scale bar, 100 μm. Data are presented as mean ± SD from three independent experiments. (* *p* < 0.05, ** p < 0.01, *** p < 0.001).

Previous studies have demonstrated that SH-SY5Y cells express high levels of GSDME 33, which can be cleaved by caspase-3 to produce GSDME-N (GSDME N-terminal fragment), a pore-forming N-terminal domain 33, 34. GSDME-N is capable of permeabilizing the mitochondrial membrane, thereby facilitating the release of cytochrome c, activating the apoptosome, and ultimately enhancing apoptosis 35. We subsequently investigated GSDME processing in cells treated with Cd and/or PQ. As illustrated in Fig. 3a-c, co-exposure to Cd and PQ gradually induced the cleavage of full-length GSDME into the ~30 kDa pore-forming fragment, GSDME-N. Notably, mitochondrial translocation of GSDME-N was significantly enhanced in co-exposed cells (Fig. 3d), which is consistent with its established role in promoting mitochondrial permeabilization. Previous study demonstrated that Pan-caspase inhibitors effectively suppress GSDME cleavage^36^. We also observed that pre-treatment with Q-VD substantially reduced GSDME-N levels (Fig. 3e). To further substantiate the critical role of GSDME in co-exposure-induced apoptosis, we utilized shRNA to knock down GSDME expression (Fig. 3f). The results revealed that GSDME knockdown markedly attenuated apoptosis induced by co-exposure to Cd and PQ (Fig. 3g, h). In addition, we also evaluated the potential contribution of GSDMD, another key executor of pyroptosis. Notably, SH-SY5Y cells with GSDMD knockdown exhibited no significant difference in morphological change or apoptotic rate compared to control cells under Cd and PQ co-exposure (Supplementary Fig.3a-c), suggesting that GSDMD is unlikely to play a functional role in this pathway. Collectively, these data demonstrate that Cd and PQ co-exposure induces apoptosis in a GSDME-dependent manner.

### Mito-ROS play a crucial role in the activation of GSDME and induction of apoptosis

**Figure 4:**
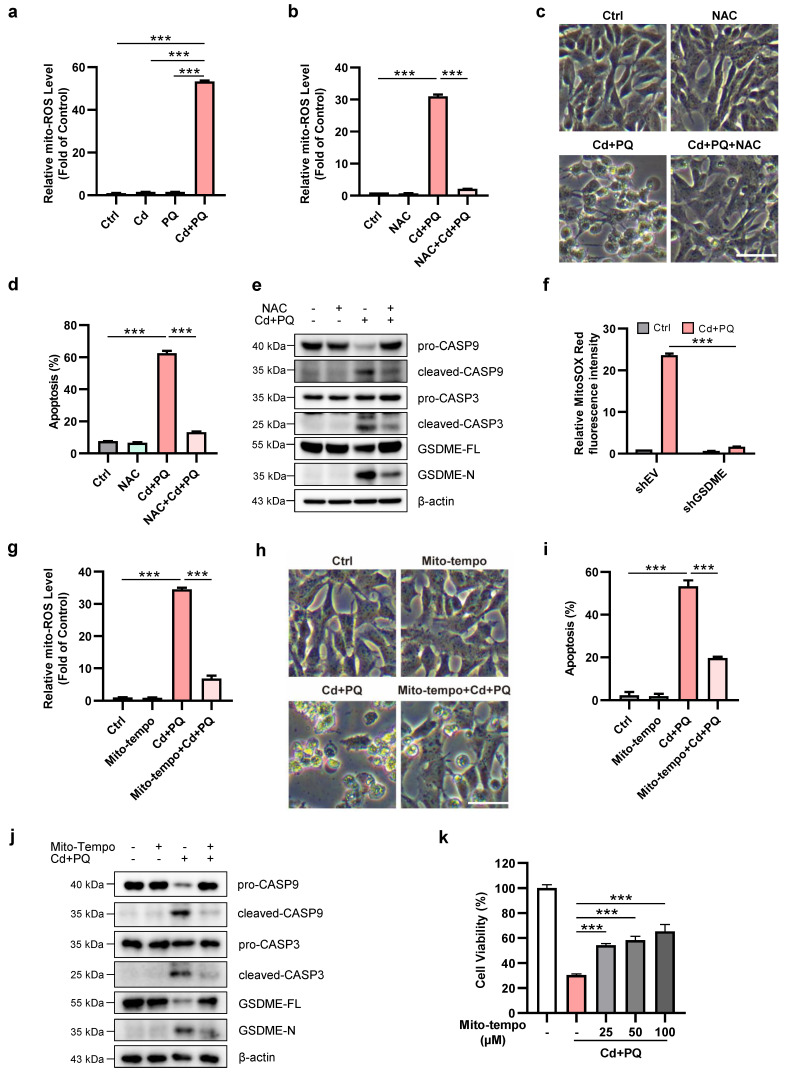
** Mitochondrial ROS play a crucial role in the activation of GSDME and induction of apoptosis. (a)** Mitochondrial ROS (mito-ROS) levels were detected by flow cytometry after 36 h co-exposure (5 μM Cd and 150 μM PQ). **(b-e)** Mito-ROS levels **(b)**, cell morphology **(c)**, apoptosis rates **(d)**, and expression of caspase-9 (CASP9)/caspase-3 (CASP3)/GSDME proteins **(e)** were assessed in SH-SY5Y cells exposed to Cd (5 μM) and PQ (150 μM) for 36 h, with or without 1 h pre-treatment with NAC (5 mM). scale bar, 100 µm. **(f)** SH-SY5Y control (shEV) and GSDME knockdown (shGSDME) cells were exposed to Cd (5 μM) and PQ (150 μM) for 36 h. mito-ROS levels were subsequently quantified by flow cytometry. **(g-j)** Mito-ROS release level **(g)**, microscopic morphology** (h)**, apoptosis rates **(i)**, and caspase-9 (CASP9)/caspase-3 (CASP3)/GSDME proteins** (j)** in SH-SY5Y cells exposed to Cd (2.5 μM) and PQ (150 μM) for 36 h, with or without 1 h pre-treatment with Mito-Tempo (100 μM). scale bar, 100 µm. **(k)** SH-SY5Y cells were pretreated with varying concentrations of Mito-Tempo for 1 h, followed by exposure to Cd (2.5 μM) and PQ (150 μM) for an additional 36 h. Cell viability was then assessed using the MTT assay. Data are presented as mean ± SD from three independent experiments. (* *p* < 0.05, ** p < 0.01, *** p < 0.001).

Mitochondria are the primary source of intracellular ROS generated during oxidative phosphorylation, and mito-ROS also function as signaling molecules involved in various cellular processes, including apoptosis and aging 37. Therefore, we investigated whether co-exposure to Cd and PQ could elevate ROS levels in SH-SY5Y cells, thereby inducing cell death. We quantified mito-ROS using MitoSOX Red staining. Our results showed that the co-exposure group displayed a significant increase in mito-ROS compared to the other groups (Fig. 4a and Supplementary Fig.4a-b). This increase was almost completely suppressed by the ROS scavenger NAC (Fig. 4b). Notably, NAC effectively suppressed apoptosis induced by co-exposure to Cd and PQ (Fig. 4c, d). Furthermore, NAC significantly inhibited the cleavage of caspase-9, caspase-3, and GSDME (Fig. 4e). We next examined whether GSDME contributed to mito-ROS generation under co-exposure conditions. Flow cytometry analysis using MitoSOX Red staining revealed a pronounced leftward shift in fluorescence intensity in GSDME-silenced cells compared to controls (Supplementary Fig.4c). This reduction in mito-ROS levels was further quantified and confirmed by fluorescence intensity analysis, showing a marked decrease in GSDME-knockdown cells (Fig. 4f). These findings suggest that mito-ROS may serve as both an initiator and amplifier of the apoptotic cascade. Specifically, oxidative stress first triggers caspase-3/GSDME activation. This leads to the insertion of GSDME-N into the mitochondrial membrane. Consequently, mitochondrial function is impaired, further exacerbating ROS production. This process establishes a self-reinforcing cytotoxic loop.

To further confirm mito-ROS as the pivotal trigger of the apoptotic cascade, we employed the specific mito-ROS scavenger Mito-Tempo. Treatment with Mito-Tempo significantly suppressed co-exposure-induced mito-ROS accumulation (Fig. 4g), accompanied by a marked reduction in neuronal apoptosis (Fig. 4h, i). Moreover, Mito-Tempo treatment effectively inhibited the cleavage of caspase-9, caspase-3, and GSDME (Fig. 4j). Consistent with these molecular changes, the MTT assay revealed that Mito-Tempo dose-dependently alleviated cytotoxicity in co-exposed cells (Fig. 4k). Collectively, these findings demonstrate that co-exposure to Cd and PQ induces apoptosis through excessive mito-ROS production.

### Mito-ROS augments mitochondrial damage via imbalance of OPA1 processing

**Figure 5:**
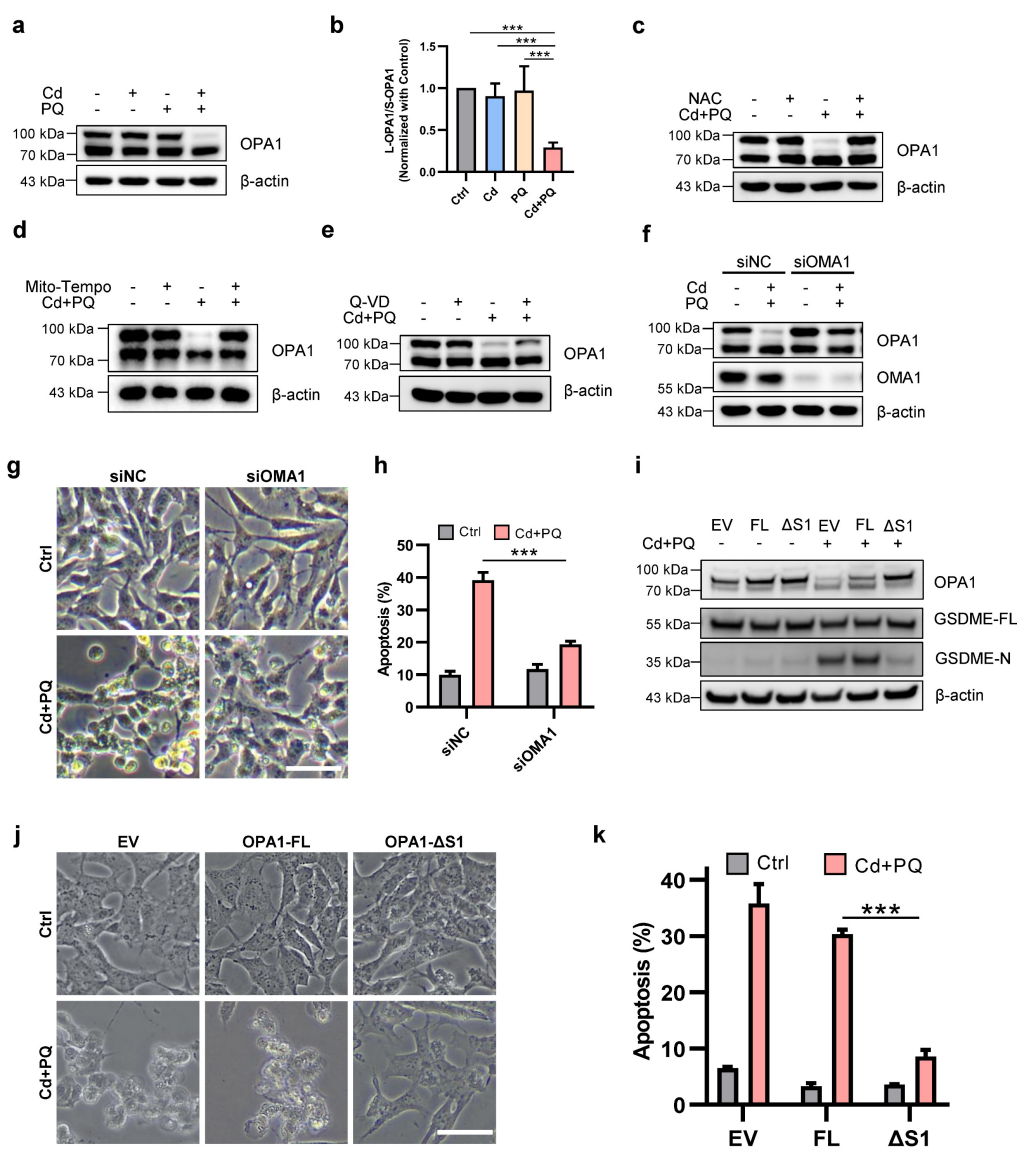
** Mitochondrial ROS augments mitochondrial damage via imbalance of OPA1 processing. (a)** Western blot analysis of OPA1 after 5 μM Cd and/or 150 μM PQ exposure for 36 h. **(b)** Quantification of the ratio of L-OPA1 to S-OPA1 (L/S-OPA1) were shown in the bar graph.** (c-e)** OPA1 levels in SH-SY5Y cells treated with Cd (5 μM) and PQ (150 μM) in the presence or absence of 1 h pre-treatment of 5 mM NAC **(c)**, 100 μM Mito-Tempo** (d)** or 20 μM Q-VD **(e)** were assayed by Western blot. **(f-h)** SH-SY5Y cells were exposed to Cd (5 μM) and PQ (150 μM) for 36 h with or without OMA1 siRNA transfection, and the OPA1 and OMA1 levels **(f)**, microscopic morphology **(g)**, apoptosis rates **(h)** in SH-SY5Y cells were evaluated. Scale bar, 100 µm.** (i-k)** SH-SY5Y cells overexpressing OPA1-FL (full-length) or OPA1-ΔS1 (S1-site cleavage-resistant) were treated with Cd (5 μM) and PQ (150 μM) for 36 h, followed by analyses of OPA1 and GSDME expression **(i),** cell morphology** (j)** and apoptosis rates** (k)**. scale bar, 100 µm. Data are presented as mean ± SD from three independent experiments. (* *p* < 0.05, ** p < 0.01, *** p < 0.001).

Given the central role of mitochondrial dysfunction in apoptosis and the pathogenesis of PD 38, we further ascertain the effects of Cd and/or PQ exposure on mitochondria homeostasis. Since mitochondria function as the "powerhouse of the cells," we first investigated whether Cd and/or PQ affect ATP production. Although single exposure did not alter ATP levels, co-exposure to Cd and PQ resulted in a significant depletion of ATP (Supplementary Fig. 5a). The structural integrity of mitochondria is crucial for maintaining mitochondrial function. We therefore utilized high-resolution microscopy to examine mitochondrial morphology. As the data indicate, co-exposure induced severe mitochondrial fragmentation, characterized by the transformation of tubular networks into large punctate structures (Supplementary Fig. 5b). The mitochondrial morphology is regulated by the dynamic balance between mitochondrial fusion and fission processes. We further examined the effects of Cd and/or PQ exposure on the expression levels of fusion-related proteins, including OPA1, mitofusin 1 and 2 (MFN1 and MFN2), as well as the fission-related protein DRP1. Notably, co-exposure to Cd and PQ significantly altered the expression of OPA1 (Fig. 5a, b). Although the total levels of mitochondrial dynamics proteins (MFN1 and MFN2) remained largely unchanged (Supplementary Fig. 5c and 5d), a slight reduction in DRP1 phosphorylation at Ser637 was observed (Supplementary Fig. 5e), an inhibitory modification known to restrain DRP1 activity and mitochondrial fission 39. This decrease in pS637-DRP1 may suggest enhanced DRP1 activation and mitochondrial fission. Together with the impaired OPA1-mediated fusion under sustained mito-ROS stress, this likely drives excessive mitochondrial fragmentation. The imbalance in mitochondrial fusion and fission underlies the severe structural damage and increased apoptotic vulnerability induced by Cd and PQ co-exposure. Further studies are needed to elucidate how co-exposure affects DRP1 activity.

OPA1 is a dynamin-related GTPase that exists in long (L-OPA1) and short (S-OPA1) isoforms due to regulated proteolytic processing. Studies have demonstrated that under stress conditions, L-OPA1 mediates mitochondrial fusion, whereas S-OPA1 promotes mitochondrial fission 40-42. Consistent with these findings, we observed that co-exposure to Cd and PQ significantly reduced L-OPA1 expression and decreased the L/S-OPA1 ratio (Fig. 5a, b and Supplementary Fig. 5f), suggesting that co-exposure impairs OPA1 processing. To further elucidate the role of OPA1 processing in neuronal cell death, we investigated its relationship with mito-ROS generation and apoptotic pathway activation. Notably, co-treatment with NAC and Mito-Tempo completely restored L-OPA1 protein expression (Fig. 5c, d), whereas co-treatment with Q-VD only partially rescued L-OPA1 levels (Fig. 5e). These findings indicate that mito-ROS serves as the predominant upstream regulator of OPA1 processing. It is well established that L-OPA1 can be cleaved into S-OPA1 at the S1 site by the inner mitochondrial membrane peptidase OMA1 or at the S2 site by the i-AAA protease YME1L 40, while the activity of OMA1 is significantly enhanced under various stress conditions 43. We thus investigate whether the degradation of L-OPA1 induced by co-exposure is dependent on OMA1. Our results demonstrated that knocking down OMA1 effectively restored L-OPA1 expression (Fig. 5f) and improved cell viability (Fig. 5g, h) in co-exposed cells. To further confirm that OMA-1-mediated OPA1 cleavage is critical for co-exposure-induced toxicity, we generated SH-SY5Y cell lines stably expressing OPA1-FL and OPA1-ΔS1. Notably, overexpression of OPA1-ΔS1, but not OPA1-FL, led to a more pronounced increase in L-OPA1 levels compared, accompanied by significant suppression of GSDME-N generation (Fig. 5i). Moreover, OPA1-ΔS1, but not OPA1-FL, effectively mitigated co-exposure-induced cell death (Fig. 5j, k). Collectively, these findings indicate that Cd and PQ co-exposure induces mitochondrial dysfunction by disrupting the balance of OPA1 processing.

Mitochondrial membrane potential (MMP) and mitophagy are other crucial indicator of mitochondrial function. However, no significant changes in MMP (Supplementary Fig. 5g) nor mitophagy (Supplementary Fig. 5h) were observed in any of the groups.

### Co-exposure to Cd and PQ induced significant neurobehavioral deficits in mice

**Figure 6:**
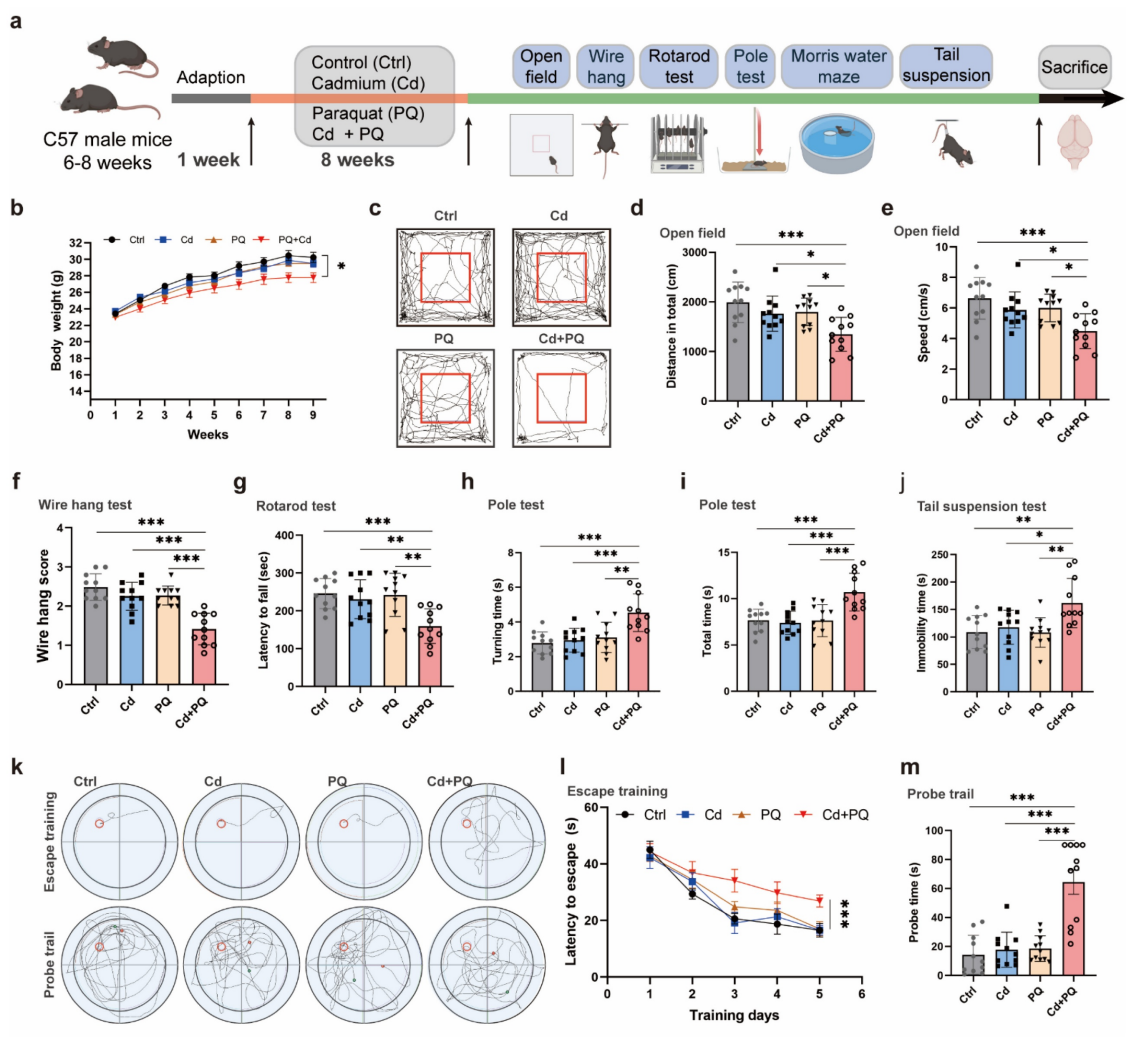
** Co-exposure to Cd and PQ induces significant neurobehavioral deficits in mice. (a)** Schematic design of the animal experiments. Male C57BL/6 mice were administered 25 mg/L Cd in drinking water and/or intraperitoneally injected with 5 mg/kg PQ (twice weekly) for 8 weeks. Afterward, behavioral tests and pathological examinations were conducted.** (b)** Body weight change curve. **(c)** Representative travel paths of mice in the open field test (OFT). **(d, e)** Total distance traveled **(d)** and mean speed **(e)** in the OFT.** (f)** Grip score measured via the wire hang test. **(g)** Time taken to fall off the rotarod. **(h, i)** Time required to descend the pole. **(j)** Immobility time in the tail suspension test. **(k)** Representative travel path tracings of mice in spatial learning and spatial memory test in the Morris water maze (MWM). **(l)** Escape latency to the hidden platform in the MWM. **(m)** Escape latency in the probe test. Data are presented as mean ± SD, n=11 mice per group. (*p < 0.05, **p < 0.01, ***p < 0.001).

To evaluate the neurotoxic effects of co-exposure to Cd and PQ, we conducted a series of animal experiments. Specifically, 6- to 8-week-old male mice were exposed to Cd and/or PQ for 8 weeks and subsequently underwent a comprehensive set of behavioral assessments (Fig. 6a). Although neither Cd nor PQ alone significantly impacted body weight gain, mice co-exposed to both substances demonstrated a substantially reduced weight gain compared to the control group and single-treatment groups (Fig. 6b). This finding suggests a synergistic adverse effect resulting from the combined exposure.

Next, to evaluate the motor deficits associated with PD-like phenotypes, we conducted a series of behavioral assessments. Spontaneous motor activity, a key indicator of PD-related motor dysfunction, was assessed using the open field test. Co-exposed mice exhibited a significant reduction in total spontaneous activity (Fig. 6c). Quantification of movement patterns revealed that both the total distance traveled (Fig. 6d) and the mean speed traveled (Fig. 6e) were markedly reduced in the co-exposure group compared to the control and single-treatment groups, suggesting more severe motor impairments. The wire hang test, which evaluates grip strength, stamina, and neuromuscular function, showed that mice in the co-exposure group had substantially lower hanging scores than all other groups (Fig. 6f). Additional motor deficits were quantified using the rotarod and pole tests. Co-exposed mice demonstrated shorter fall times in the rotarod test (Fig. 6g) and longer descent times in the pole test (Fig. 6h, i), further confirming PD-like motor abnormalities. The tail suspension test, which measures stress-induced motor immobility, indicated an increased duration of immobility in co-exposed mice (Fig. 6j), consistent with the observed motor dysfunction.

Furthermore, to evaluate the impact of co-exposure to Cd and PQ on cognitive function, a MWM test was performed. During the acquisition phase, mice co-exposed to Cd and PQ exhibited significantly prolonged escape latencies compared with other groups (Fig. 6k, l). In the spatial probe trial, co-exposed mice spent less time in the target quadrant and spent more time to first reach the escape platform, suggesting impaired spatial memory (Fig. 6k, m).

Collectively, these* in vivo* findings indicate that chronic co-exposure to environmentally relevant low doses of Cd and PQ synergistically induces PD-like neurobehavioral deficits in mice.

### Co-exposure to Cd and PQ induced nigrostriatal dopaminergic neurodegeneration

**Figure 7:**
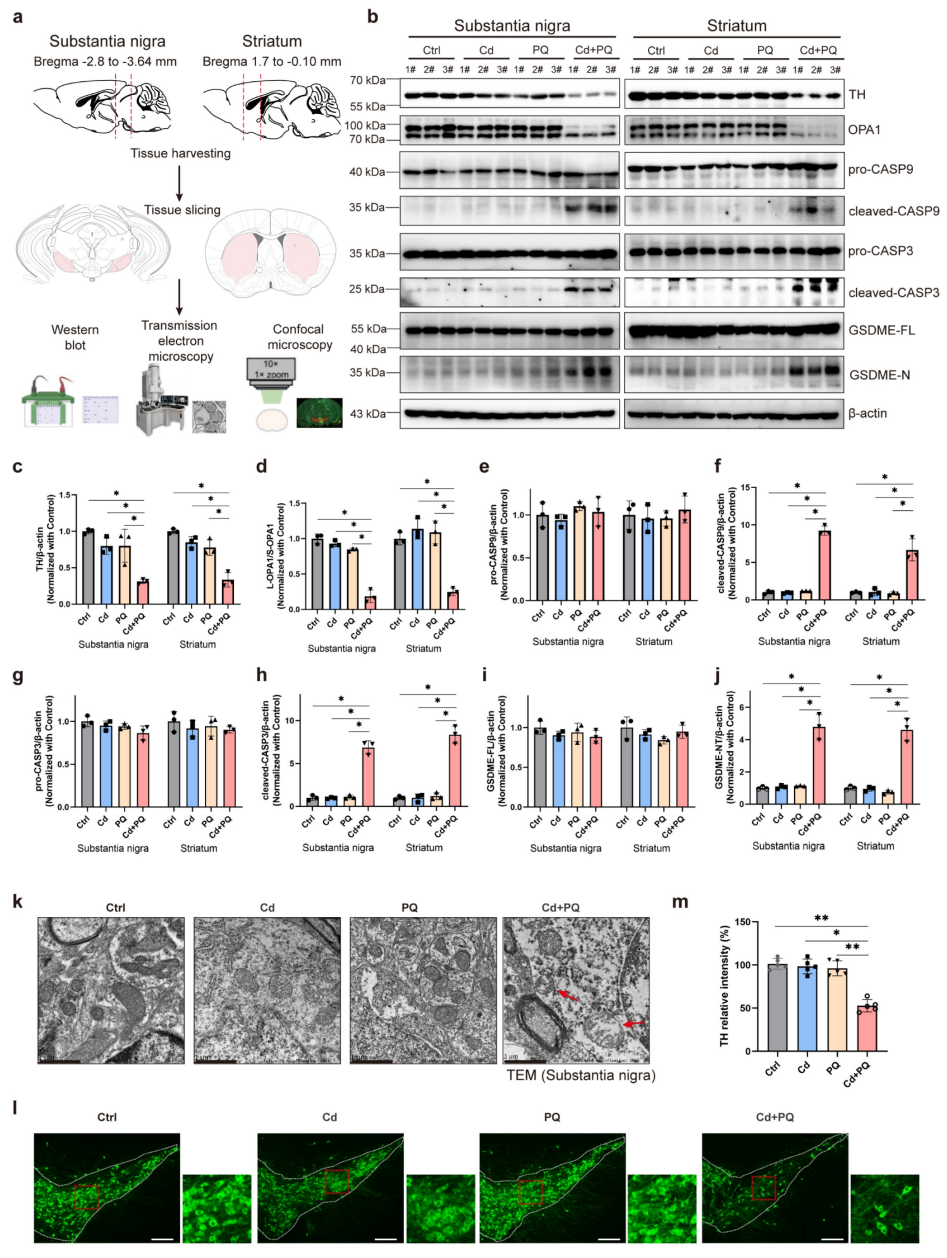
** Co-exposure to Cd and PQ induced nigrostriatal dopaminergic neurodegeneration. (a)** After treatment, the mice brains were collected via craniotomy, and the substantia nigra (SN) and striatum (STR) were isolated for further experimentation.** (b-j)** Representative blots and quantification showing the expressions of TH, OPA1, caspase-9 (CASP9), caspase-3 (CASP3) and GSDME in the SN and STR. Data are presented as mean ± SD, n=3. (*p < 0.05, **p < 0.01, ***p < 0.001.) **(k)** Representative mitochondrial morphology in SN obtained by transmission electron microscopy. Scale bar, 1 μm. **(l)** Representative immunofluorescence labeling for TH in the SN. Scale bar, 200 μm. Higher magnification views are shown on the right side. **(m)** The relative TH intensity in SN (n=5). (*p < 0.05, **p < 0.01, ***p < 0.001).

PD is characterized by progressive motor and non-motor dysfunctions, largely attributed to the degeneration of dopaminergic neurons within the midbrain nigrostriatal system. To evaluate the neurotoxic effects of Cd and PQ co-exposure on dopaminergic neuronal loss, we dissected the bilateral SN and STR for molecular and morphological analysis (Fig. 7a). TH, a rate-limiting enzyme in dopamine synthesis and a canonical marker of dopaminergic neurons, was significantly downregulated in both SN and STR tissues of co-exposed mice (Fig. 7b, c). Notably, the OPA1-mediated caspase-9/caspase-3/GSDME apoptotic pathway previously identified in SH-SY5Y cells was recapitulated *in vivo*. Specifically, co-exposure resulted in a marked reduction in the L-OPA1/S-OPA1 ratio in both SN and STR tissues (Fig. 7b and 7d), along with increased levels of activated apoptotic effectors, including cleaved caspase-9, cleaved caspase-3, and GSDME-N, thereby confirming the involvement of this apoptotic pathway in dopaminergic neuronal death (Fig. 7b and 7e-j).

Given that disrupted OPA1 processing is closely associated with mitochondrial fragmentation 40-42, we further investigated the mitochondrial ultrastructure in SN dopaminergic neurons using transmission electron microscopy. Mice co-exposed to Cd and PQ exhibited severe mitochondrial abnormalities compared to other groups, including outer membrane rupture, cristae disorganization or loss, and mitochondrial swelling (Fig. 7k and Supplementary Fig. 6; red arrows indicate damaged mitochondria). These morphological alterations highlight the detrimental effects of co-exposure on mitochondrial integrity. Immunofluorescence staining for TH in the SN further revealed a significant decrease in TH-positive neurons in the co-exposure group (Fig. 7l, m). Collectively, these findings indicate that chronic co-exposure to Cd and PQ aberrantly activates the OPA1/caspase-3/GSDME signaling pathway in the nigrostriatal system. This activation leads to mitochondrial dysfunction and caspase-dependent apoptosis. Consequently, progressive nigrostriatal degeneration occurs, recapitulating key neuropathological features of PD.

## Discussion

**Figure 8:**
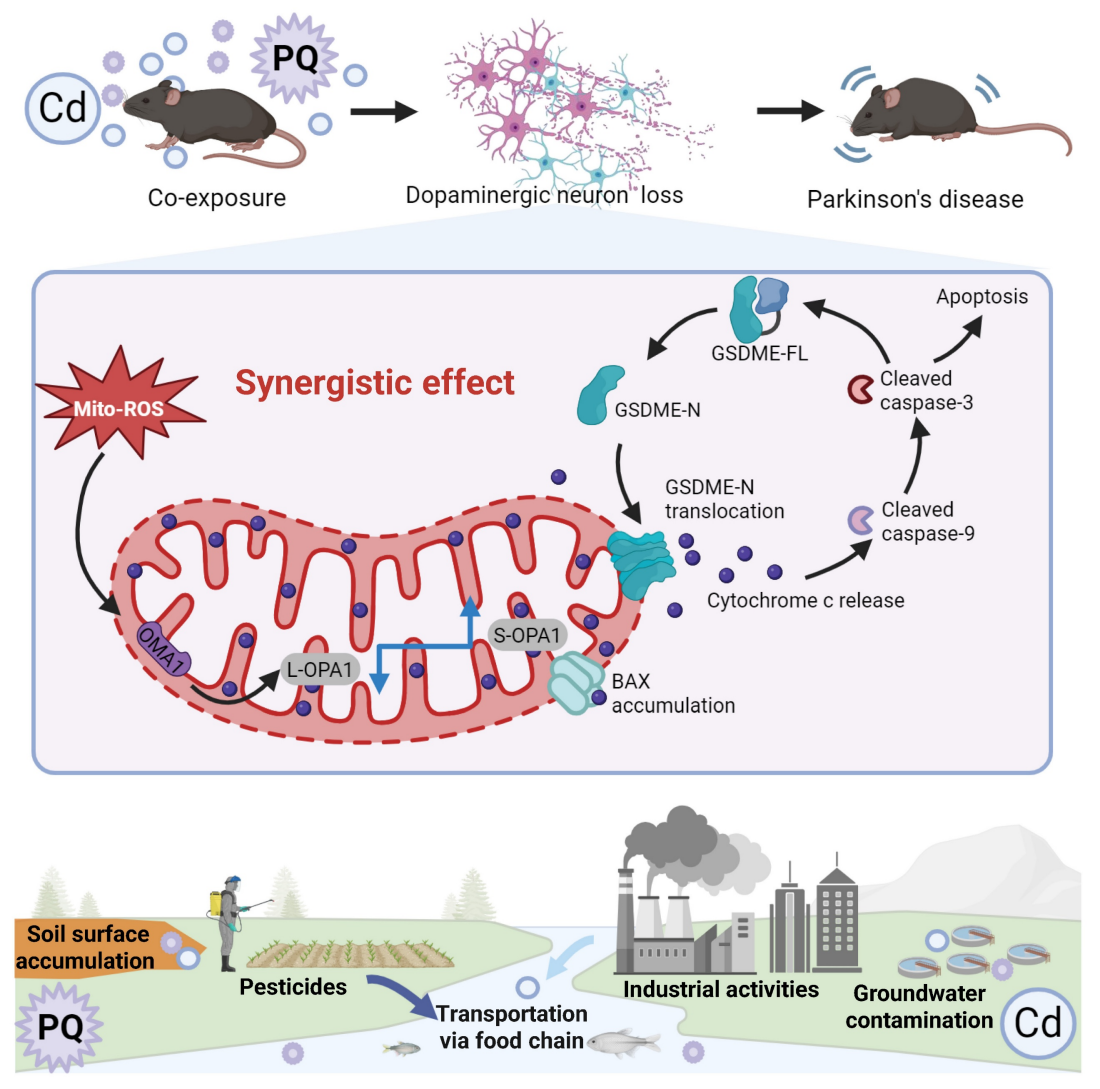
** Cd/PQ co-exposure triggers parkinsonism-like neurotoxicity through mito-ROS/OPA1/Caspase-3/GSDME pathway.** Co-exposure to Cd and PQ increases mito-ROS production, disrupts OPA1 processing, and activates the caspase cascade and GSDME, leading to apoptosis, neuron loss, and motor and cognitive impairments.

As the global population ages, PD has emerged as one of the fastest-growing neurological disorders, associated with the highest rates of disability and mortality in the past two decades 44. While the exact etiology of PD remains unclear, environmental factors such as pesticides and heavy metals have been identified as potential contributors to its increased incidence 45. In natural environments, individuals are often concurrently exposed to multiple hazardous substances, including Cd and PQ, which are commonly found in air, soil, and water. However, to date, no studies have systematically investigated the effects of co-exposure to subtoxic levels of Cd and PQ on neuronal damage or elucidated the underlying mechanisms. In this study, we demonstrate that co-exposure to Cd and PQ induces synergistic cytotoxicity and contributes to PD-like symptoms via the mito-ROS/OPA1/caspase-3/GSDME signaling pathway (Fig. 8). Mechanistically, co-exposure enhances mito-ROS production, leading to mitochondrial fragmentation by disrupting OPA1 processing. This process is followed by BAX activation, cytochrome c release, and caspase cascade activation. Subsequently, active caspase-3 cleaves GSDME to generate GSDME-N, which translocate to mitochondria and exacerbates mitochondrial damage, ultimately inducing neuronal apoptosis. Furthermore, *in vivo* PD models provide robust evidence that co-exposure triggers nigrostriatal dopaminergic neurodegeneration and neurobehavioral deficits.

Exposure to environmental pollutants cause neuronal cell death, which contributes to the pathology of PD. Consistent with previous studies 46, 47, we found that Cd or PQ exposure induced neurotoxicity in a dose-dependent manner (Fig. 1a-h and Supplementary Fig. 1a, b). Importantly, we observed that co-exposure to subtoxic Cd and PQ significantly enhanced their cytotoxicity and induced pronounced cell death (Fig. 1i-k and Supplementary Fig. 1c-e), suggesting a synergistic role of Cd and PQ in promoting PD pathogenesis. Studies have shown that programmed cell death including apoptosis, pyroptosis, necroptosis, and ferroptosis, contribute differentially to PD progression 48. Our findings reveal that neuronal cell death induced by co-exposure to Cd and PQ predominantly corresponded to apoptosis, a conclusion supported by substantial evidence including increased Annexin V-FITC^+^/PI^+^ populations, DNA fragmentation, and sequential cleavage of caspase-9/caspase-3 (Fig. 2a-d). In addition, co-exposure triggered mitochondrial translocation of BAX and cytochrome c release, confirming mitochondrial commitment to intrinsic apoptosis initiation (Fig. 2e-i). Furthermore, only the pan-caspase inhibitor Q-VD, rather than ferroptosis or necroptosis inhibitors, was able to rescue the cell death induced by co-exposure (Fig. 2j-l and Supplementary Fig. 2). In this study, Cd was administered via drinking water to mimic chronic human exposure via contaminated water and food 49, 50. Meanwhile, PQ was delivered intraperitoneally to ensure consistent systemic bioavailability, in line with established PD modeling methods 51, 52. Although these routes differ in their initial absorption and distribution kinetics, both contaminants eventually reach the systemic circulation and accumulate in neural tissues. This approach mimics the actual convergence of these pollutants within the central nervous system, reflecting real-world scenarios where humans are concurrently exposed to environmental Cd (primarily via ingestion) and PQ (through multiple routes such as inhalation, dermal contact, and ingestion). Despite these route-dependent kinetic differences, this low dose, chronic co exposure model captures relevant pathophysiological interactions and provides a framework for studying their synergistic effects at mitochondrial and cellular levels.

GSDME is a critical member of the gasdermin protein family, initially identified as causing familial hereditary deafness when mutated 53. Subsequent studies have demonstrated that GSDME plays a pivotal role in the regulation and conversion between apoptosis and pyroptosis 33, 34, 54. For instance, GSDME-N can translocate to mitochondria, increase mitochondrial membrane permeability, release cytochrome c, activate the apoptosome, and subsequently trigger caspase-3 activation, thereby inducing cell apoptosis. Activated caspase-3 further cleaves GSDME, generating additional GSDME-N, which exacerbates mitochondrial damage and reinforces the apoptotic process 35. Consistently, our findings demonstrate that GSDME served as a critical amplifier of neuronal apoptosis induced by co-exposure to Cd and PQ. Specifically, co-exposure, rather than single exposure, markedly promoted the cleavage of full-length GSDME into its pore-forming fragment, GSDME-N. GSDME-N subsequently translocated to mitochondria and synergized with BAX to induce membrane permeabilization. This process further exacerbated cytochrome c release (Fig. 3a-e). This event leads to the activation of the caspase cascade and establishes a pathogenic positive feedback loop for apoptosis, as previously reported 35. Furthermore, the functional significance of GSDME was validated through shRNA-mediated knockdown experiments, in which GSDME silencing markedly reduced apoptosis induced by co-exposure (Fig. 3f-h). Notably, classical features of pyroptosis, such as plasma membrane ballooning and rapid cell lysis, were not observed. Instead, cells displayed typical apoptotic morphology, including cellular shrinkage and nuclear condensation. Moreover, pharmacological inhibition of necroptosis or ferroptosis did not alleviate Cd plus PQ-induced cytotoxicity (Supplementary Fig. 2), while inhibition of apoptosis significantly reduced cell death. These findings imply that under conditions of persistent mitochondrial dysfunction and predominant apoptotic signaling, GSDME-N preferentially targets mitochondria to amplify apoptosis rather than forming plasma membrane pores to trigger pyroptosis. Similarly, other gasdermin family members, such as GSDMA and GSDMD, have also been reported to activate apoptosis through mitochondrial or endoplasmic reticulum stress 55, 56. These findings suggest that although gasdermin family members are conventionally defined by their role in pyroptosis, under specific stimuli or in distinct cellular contexts, gasdermins can also regulate alternative modes of cell death, including apoptosis.

Oxidative stress serves as a unifying mechanism underlying environmental neurotoxicity and the pathogenesis of PD. Both Cd and PQ independently disrupt redox homeostasis. Cd achieves this via NF-κB/AP-1-mediated pro-oxidant signaling and activation of the mTOR/Erk1/2 pathway 57, while PQ induces oxidative damage through free radical production 10 and inhibition of mitochondrial complex I 27. Mito-ROS act as critical upstream regulators of intrinsic apoptosis. For example, TP3-induced osteosarcoma cell apoptosis 58 and Magnolol-mediated neuroblastoma cell death 59 both rely on mito-ROS-dependent caspase activation. In our model, co-exposure-induced mito-ROS overproduction, neuronal cell death, and the expression of cleaved caspase-3 and GSDME-N were significantly reduced following treatment with ROS scavengers NAC or Mito-Tempo (Fig. 4). These findings indicate that mito-ROS serve as the primary mediator driving Cd and PQ co-exposure-enhanced intrinsic apoptosis.

Damaged mitochondria serve as a primary source of intracellular mito-ROS, impelling us to examine the impact of Cd and PQ co-exposure on mitochondrial function. Consistent with our hypothesis, co-exposure induced severe mitochondrial damage and ATP depletion (Supplementary Fig. 5a, b). Although cells generally mitigate such stress through mitophagy—a mitochondrial quality control mechanism dependent on MMP 60. Intriguingly, we observed severe mitochondrial ultrastructural damage without concomitant activation of mitophagy (Supplementary Figs. 5g, h). This disconnect could arise from several factors. First, chronic low-level damage in this co-exposure model might trigger subtle or delayed mitophagic responses that were not detected at the endpoint of our measurement. Second, the specific nature of Cd/PQ-induced damage (cristae disorganization without severe membrane depolarization) may not generate sufficiently strong canonical signals (such as loss of MMP) to recruit autophagy machinery. Third, Cd and PQ co-exposure may selectively disrupt key mitophagy regulators or signaling thresholds required to initiate PINK1/Parkin-dependent or NIX-dependent mitophagy, resulting in the accumulation of dysfunctional mitochondria. Further investigation into upstream signaling and flux assays would clarify this point. These findings indicate that impaired mitochondrial clearance might exacerbate oxidative injury and enhance the synergistic cytotoxicity associated with Cd and PQ co-exposure. A critical regulator of mitochondrial function is the proteolytic processing of OPA1, a mitochondrial fusion protein that is essential for maintaining cristae integrity 61, 62. Duan et al. 27 demonstrated that PQ induces mitochondrial fragmentation by disrupting the balance of OPA1 processing, thereby leading to PD-like neuropathology. In this study, our data demonstrated that Cd and PQ co-exposure-induced mito-ROS activated OMA1-mediated processing of OPA1, converting L-OPA1 into S-OPA1 (Fig. 5a-e). Furthermore, the pathological significance of OPA1 dysregulation was substantiated by OMA1 knockdown and enzyme activity mutation experiments, which restored L-OPA1 levels and rescued cell viability in co-exposed cells (Fig. 5f-k). The maintenance of healthy mitochondrial networks depends on a finely coordinated balance among biogenesis, dynamics, repair mechanisms, and degradation pathways such as mitophagy 63. Further investigations are warranted to clarify the mitochondrial biogenesis and repair mechanisms underlying co-exposure-induced cell death.

PD is a neurodegenerative disorder marked by the progressive loss of dopaminergic neurons in the SN. Its core motor symptoms include bradykinesia (slowed movement), muscle rigidity, and resting tremor. Furthermore, non-motor symptoms such as sleep disturbances, mood alterations, and cognitive decline frequently precede motor manifestations, with cognitive impairment affecting more than 80% of patients in advanced stages 64. Based on the aforementioned *in vitro* cell culture findings, we further investigated whether co-exposure to Cd and PQ exacerbates PD-like neuropathology *in vivo*. As anticipated, mice co-exposed to Cd and PQ demonstrated progressive motor deficits and cognitive impairments, as evidenced by a series of behavioral assessments, including the open field test, wire hang test, rotarod test, pole test, tail suspension test, and MWM test (Fig. 6). Mechanistically, consistent with our *in vitro* observations, in both SN and STR tissues co-exposure induced mitochondrial damage, disrupted OPA1 processing, activated the caspase cascade, and promoted GSDME-N generation (Fig. 7). These events ultimately led to dopaminergic neuron apoptosis and progressive nigrostriatal degeneration. Together, these pathological changes recapitulate the neuropathology of PD.

One limitation of the current study is the sole utilization of male mice in the *in vivo* experiments. Given the extensively documented sex-related disparities in the prevalence, progression, and pathophysiology of PD, the findings of this study may not be fully generalizable to females. In future studies, it would be worthwhile to incorporate female animal models to investigate whether the mito-ROS/OPA1/caspase-3/GSDME pathway functions in a sex-dependent manner and to explore potential sex-specific neuroprotective approaches.

## Conclusion

This study demonstrates that chronic co-exposure to subtoxic levels of Cd and PQ synergistically exacerbates PD-like neuropathology in mice. Compared with single-agent exposure, combined treatment significantly amplified mito-ROS overproduction. This enhancement exacerbated the disruption of OPA1 processing and drove caspase-3-mediated GSDME cleavage, resulting in neuronal apoptosis and nigrostriatal degeneration—hallmarks of PD. Notably, targeted modulation of the mito-ROS/OPA1/caspase-3/GSDME axis effectively attenuated these apoptotic processes. These findings not only elucidate a novel mechanism by which co-exposure to environmental toxins drives PD progression but also underscore the critical role of cumulative toxicant interactions in neurodegeneration, providing a framework for refining risk assessment strategies for mixed environmental hazards.

## Supplementary Material

Supplementary figures.

## Data Availability

All data generated or analyzed during this study are included within the article.

## Abbreviations

CASP3: caspase-3
CASP9: caspase-9
Cd: cadmium
CI: Combination Index
Fa: fractional effect
Fer-1: ferrostatin-1
GSDME-FL: full-length GSDME
GSDME-N: N-terminal fragment
L-OPA1: long OPA1
mito-ROS: mitochondrial reactive oxygen species
MMP: mitochondrial membrane potential
MWM: Morris water maze
NAC: N-acetyl-L-cysteine
Nec-1: necrostatin-1
Nec-1s: necrostatin-1s
OPA1-FL: full-length OPA1
OPA1-ΔS1: OPA1 with cleavage-resistant mutant harboring a defective S1 site
PD: Parkinson's disease
PQ: paraquat
PI: propidium iodide
ROS: reactive oxygen species
SD: standard deviation
siRNA: Small interfering RNA
SN: substantia nigra
S-OPA1: short OPA1
STR: striatum
STS: Staurosporine
TH: Tyrosine hydroxylase
