# Supplementary material for: Synergistic Neurotoxicity of environmental Cadmium and Paraquat in Parkinsonism: Unveiling the Mito-ROS/OPA1/Caspase-3/GSDME-driven Apoptosis Axis
Source: Int J Biol Sci. 2026 Feb 18;22(6):2885–905. doi: 10.7150/ijbs.126979 (PMC13050437; doi:10.7150/ijbs.126979)

Fig. S1. Effects of Cd and PQ exposure on cell death. SH-SY5Y cells were either treated with Cd **(a)** or PQ **(b)** for 36 h, and flow cytometry analysis was conducted using PI staining. **(c)** Combination index (CI) analysis of Cd + PQ interaction after 36 h. The x-axis indicates the fractional effect (Fa, proportion of affected cells), and the y-axis shows the corresponding CI value. The dashed horizontal line at CI = 1.0 denotes the additive effect threshold. Data points with CI > 1 (blue) represent antagonism, whereas points with CI < 1 (red) indicate synergy. The combination tested in this study (5 μM Cd + 150 μM PQ for 36 h) is highlighted with a green circle. **(d, e)** SH-SY5Y cells were treated with Cd (5 μM) and/or PQ (150 μM) for 36 h, showing cell proliferation **(d)** and PI uptake **(e)**. Data are presented as mean ± SD, n=3. (*$p < 0.05$, **$p < 0.01$, ***$p < 0.001$.)

Fig. S2. Effects of Cd and PQ exposure on ferroptosis and necroptosis. SH-SY5Y cells were either pretreated with 10 μM ferroptosis inhibitor (ferrostatin-1, Fer-1), or 10 μM necroptosis inhibitor (Necrostatin-1, Nec-1) or 10 μM necroptosis inhibitor (Necrostatin-1s, Nec-1s) for 1 h before treatment with Cd (5 μM) and PQ (150 μM) for 36 h. The microscopic morphology (**a, e**), Cell viability (**b**) and PI uptake **(c, d, f)** in SH-SY5Y cells were evaluated. Scale bar, 100 μm. Data are presented as mean ± SD, n=3. (ns, not significant, $p > 0.05$)

Fig. S3. GSDMD knockdown does not alter Cd and PQ-induced apoptosis in SH-SY5Y cells. (a) The efficiency of the GSDME knockdown was confirmed by Western blot. **(b)** Representative bright-field images of control siRNA (siNC) and GSDMD knockdown (siGSDMD) SH-SY5Y cells treated with Cd (5 μM) and PQ (150 μM) for 36 h. Scale bar, 100 μm. **(c)** Flow cytometric analysis of apoptosis in siNC and siGSDMD cells after 36 h of Cd (5 μM) and PQ (150 μM) exposure. Data are presented as mean ± SD, n=3. (ns, not significant, $p > 0.05$)

Fig. S4. Cd and PQ co-exposure on mito-ROS production in SH-SY5Y cells. (a) Representative flow cytometry images showing mito-ROS levels detected by MitoSOX

Red in SH-SY5Y cells after 12, 24, and 36 h of treatment with Cd (5 μM) and PQ (150 μM). **(b)** Quantification of mean fluorescence intensity derived from the flow cytometry analysis in **(a)**. Data are presented as mean ± SD (n = 3). ***$p < 0.001$. **(c)** SH-SY5Y control (shEV) and GSDME knockdown (shGSDME) cells were exposed to Cd (5 μM) and PQ (150 μM) for 36 h. Mito-ROS levels were subsequently quantified by flow cytometry.

Fig. S5. Cd and PQ co-exposure impairs mitochondrial function in SH-SY5Y cells. (**a**) Measurement of ATP levels in SH-SY5Y cells after treated with Cd (5 μM) and/or PQ (150 μM) for 36 h. (**b**) Representative confocal images of mitochondrial morphology from SH-SY5Y cells after Cd (5 μM) and/or PQ (150 μM) treatment for 36 h. Mitochondria were labeled with an anti-TOM20 antibody. Scale bar, 20 μm. (**c-e**) Representative blots showing the expressions of MFN1 (**c**), MFN2 (**d**) and DRP1 (**e**) in the SH-SY5Y cells after Cd (5 μM) and/or PQ (150 μM) exposure for 36 h. (**f**) Quantification of the L-OPA1, S-OPA1 and total-OPA1 in Fig.5a were shown in the bar graph. (**g**) The mitochondrial membrane potential levels of SH-SY5Y cells treated with Cd (5 μM) and/or PQ (150 μM) for 36 h were evaluated by MitoTracker Red CMXRos. (**h**) The detection of mitophagy using flow cytometry of SH-SY5Y cells stably expressing mito-keima treated with Cd (5 μM) and/or PQ (150 μM) for 36 h. Data are presented as mean ± SD, n=3 for (a) and n=4 for (f). (ns, not significant, *$p < 0.05$, **$p < 0.01$, ***$p < 0.001$.)

Fig. S6. Quantification of damaged mitochondria in substantia nigra dopaminergic neurons. The percentage of mitochondria exhibiting ultrastructural damage was calculated from randomly selected fields of view in transmission electron microscopy images. Data are presented as mean ± SD, n=10. (**$p < 0.01$).

Fig. S1

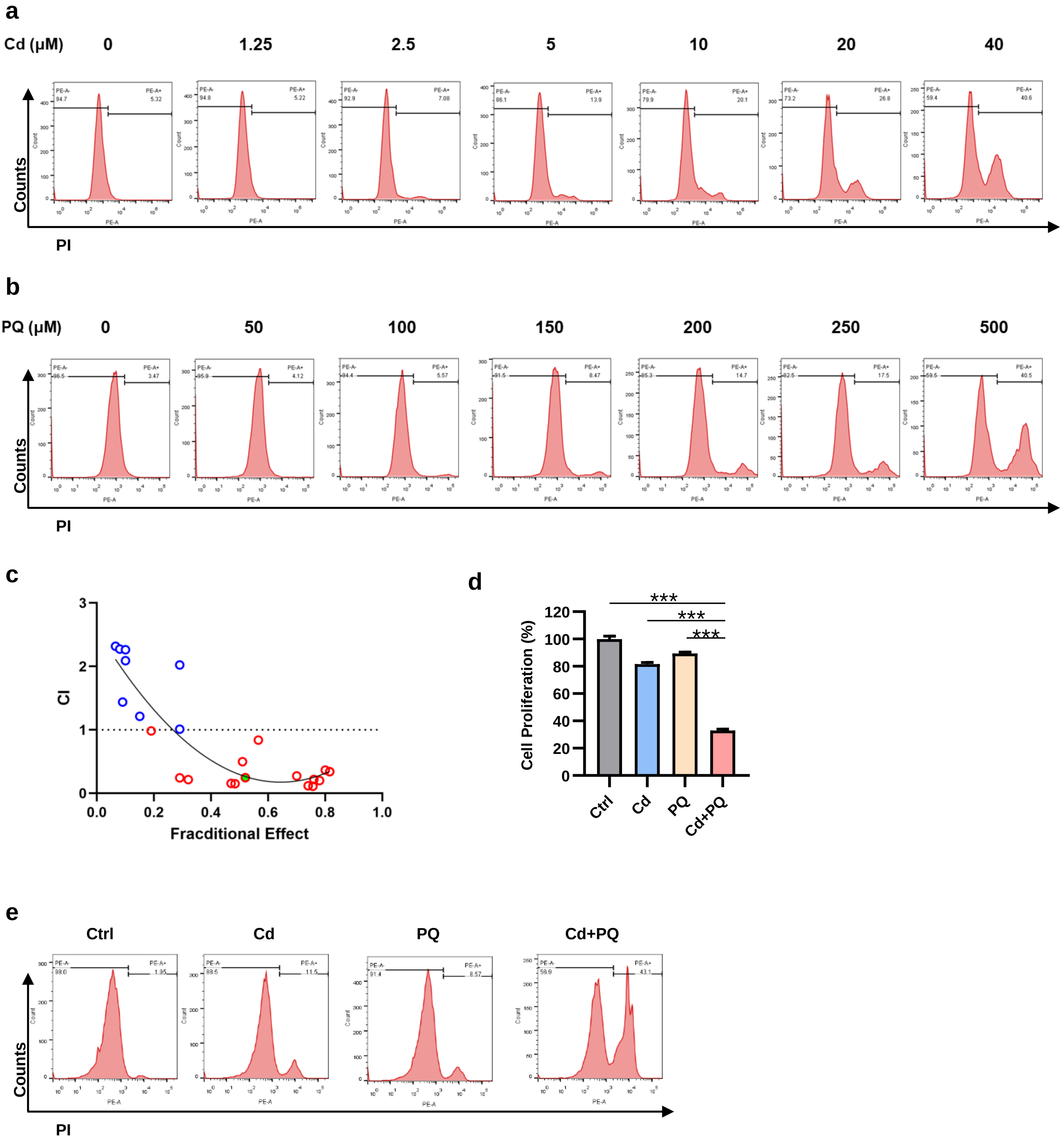
a
Cd (μM)
0
1.25
2.5
5
10
20
40
Counts
PI
b
PQ (μM)
0
50
100
150
200
250
500
Counts
PI
c
CI
Fracditional Effect
d
Cell Proliferation (%)
***
***
***
Ctrl
Cd
PQ
Cd+PQ
e
Ctrl
Cd
PQ
Cd+PQ
Counts
PI

Fig. S2

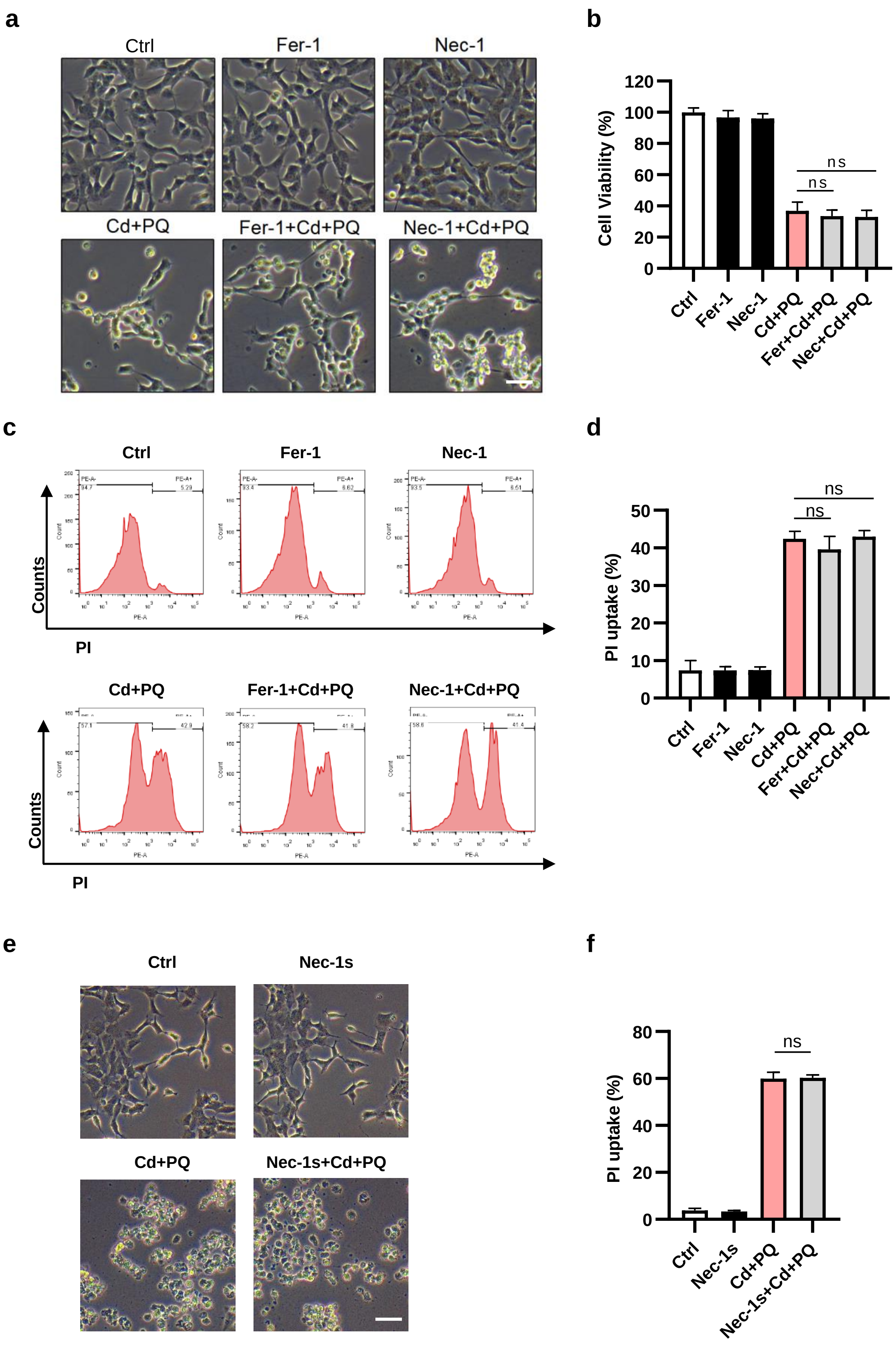
a
Ctrl
Fer-1
Nec-1
Cd+PQ
Fer-1+Cd+PQ
Nec-1+Cd+PQ
b
Cell Viability (%)
120
100
80
60
40
20
0
ns
ns
Ctrl
Fer-1
Nec-1
Cd+PQ
Fer+Cd+PQ
Nec+Cd+PQ
c
Ctrl
Fer-1
Nec-1
Counts
PI
Cd+PQ
Fer-1+Cd+PQ
Nec-1+Cd+PQ
Counts
PI
d
PI uptake (%)
50
40
30
20
10
0
ns
ns
Ctrl
Fer-1
Nec-1
Cd+PQ
Fer+Cd+PQ
Nec+Cd+PQ
e
Ctrl
Nec-1s
Cd+PQ
Nec-1s+Cd+PQ
f
PI uptake (%)
80
60
40
20
0
ns
Ctrl
Nec-1s
Cd+PQ
Nec-1s+Cd+PQ

Fig. S3

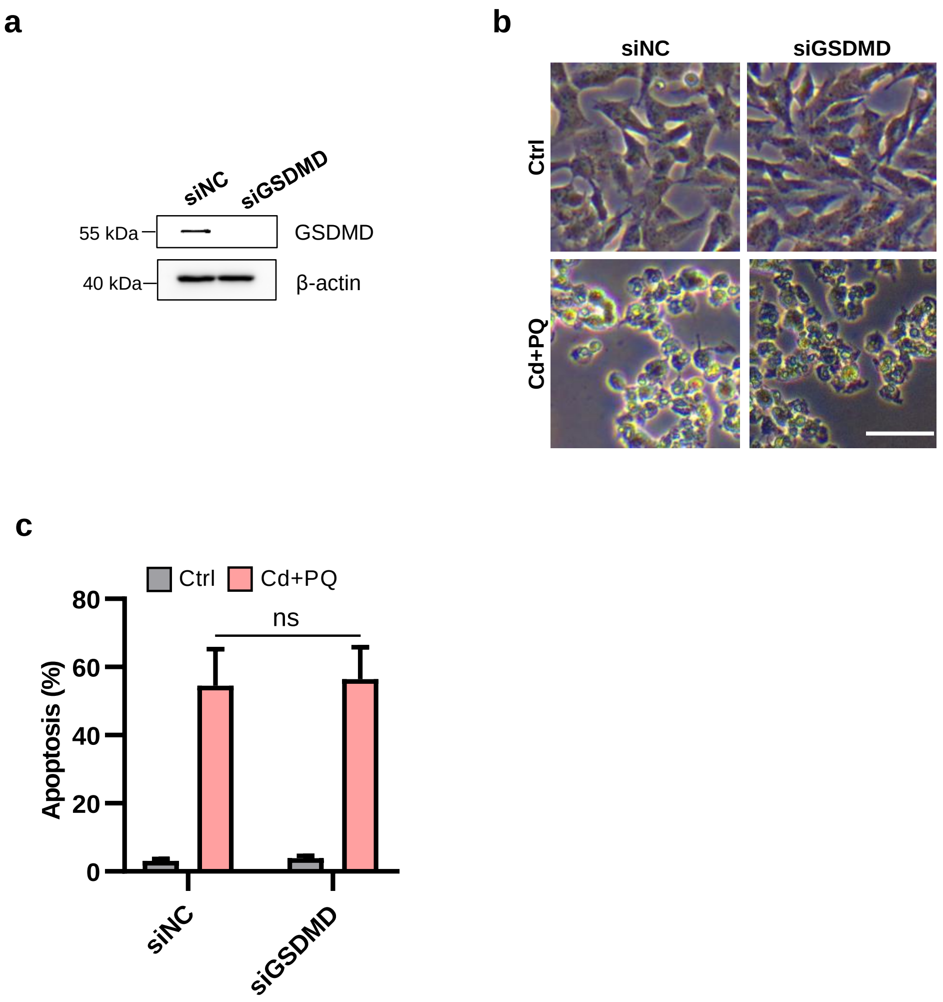
a
siNC
siGSDMD
55 kDa
GSDMD
40 kDa
β-actin
b
siNC
siGSDMD
Ctrl
Cd+PQ
c
Ctrl
Cd+PQ
80
60
40
20
0
Apoptosis (%)
ns
siNC
siGSDMD

Fig. S4

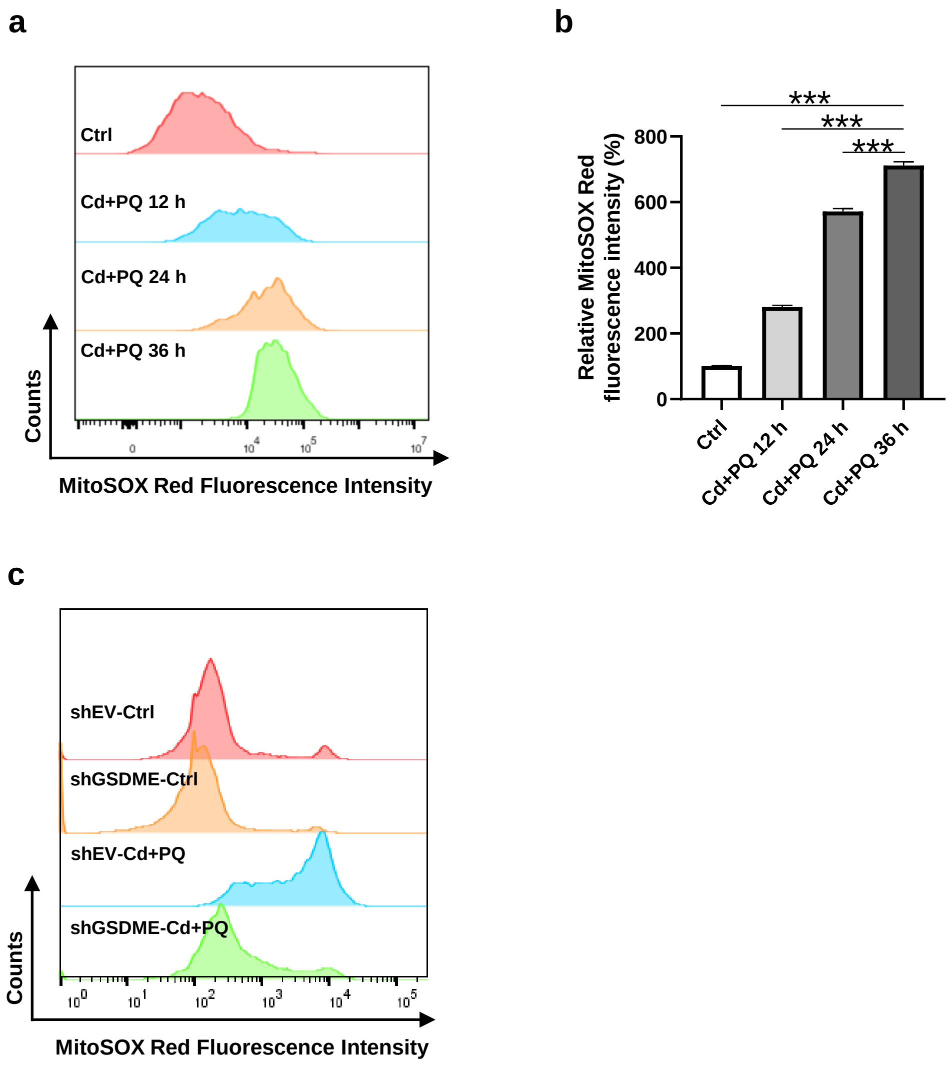
a
Ctrl
Cd+PQ 12 h
Cd+PQ 24 h
Cd+PQ 36 h
Counts
MitoSOX Red Fluorescence Intensity
b
Relative MitoSOX Red fluorescence intensity (%)
800
600
400
200
0
***
***
***
Ctrl
Cd+PQ 12 h
Cd+PQ 24 h
Cd+PQ 36 h
c
shEV-Ctrl
shGSDME-Ctrl
shEV-Cd+PQ
shGSDME-Cd+PQ
Counts
MitoSOX Red Fluorescence Intensity

Fig. S5

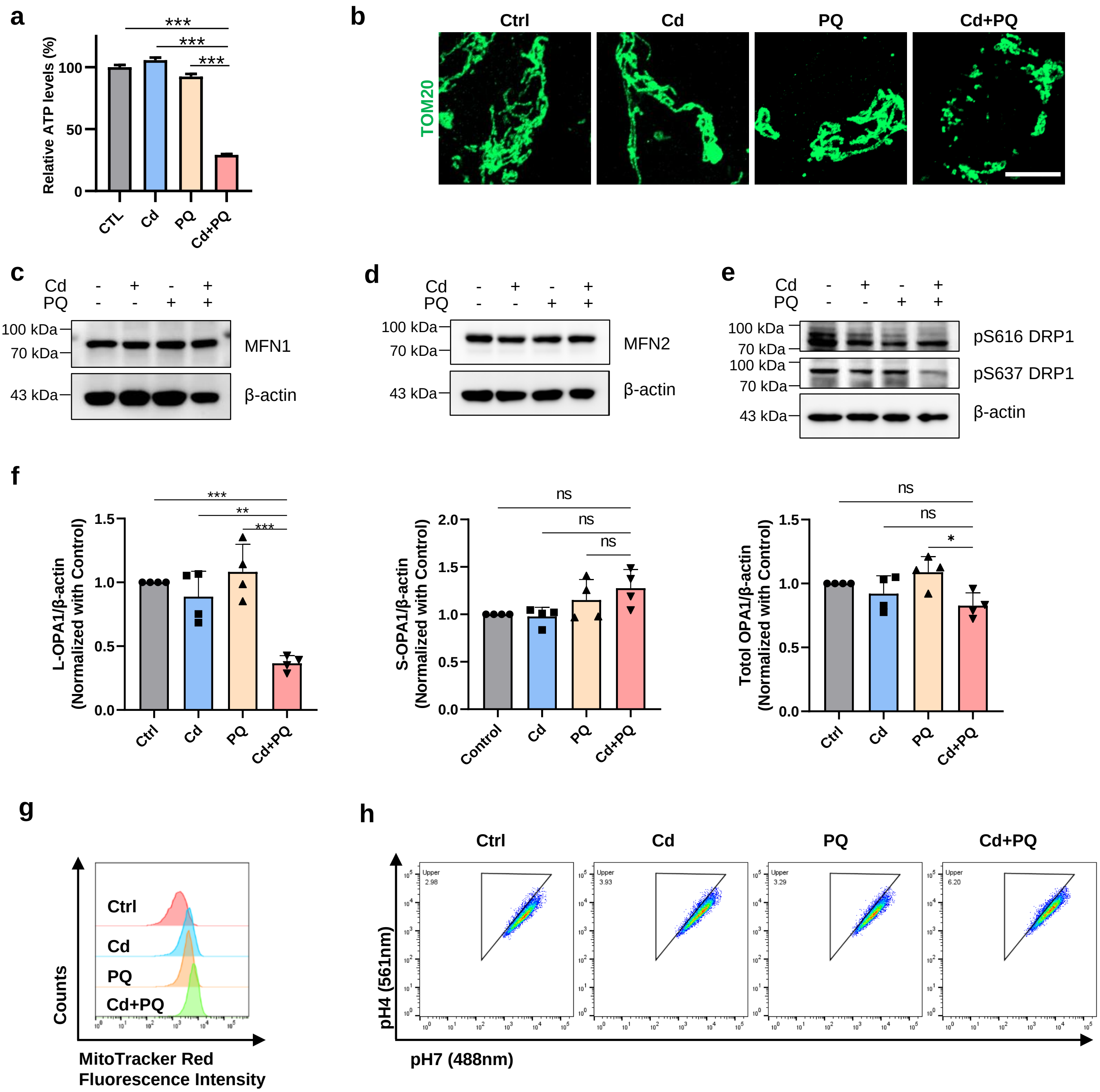

a
Relative ATP levels (%)
***
***
***
100
50
0
CTL
Cd
PQ
Cd+PQ
b
Ctrl
Cd
PQ
Cd+PQ
TOM20
c
Cd - + - +
PQ - - + +
100 kDa
70 kDa
MFN1
43 kDa
β-actin
d
Cd - + - +
PQ - - + +
100 kDa
70 kDa
MFN2
43 kDa
β-actin
e
Cd - + - +
PQ - - + +
100 kDa
70 kDa
pS616 DRP1
100 kDa
70 kDa
pS637 DRP1
43 kDa
β-actin
f
L-OPA1/β-actin
(Normalized with Control)
***
**
***
1.5
1.0
0.5
0.0
Ctrl
Cd
PQ
Cd+PQ
S-OPA1/β-actin
(Normalized with Control)
ns
ns
ns
2.0
1.5
1.0
0.5
0.0
Control
Cd
PQ
Cd+PQ
Totol OPA1/β-actin
(Normalized with Control)
ns
ns
*
1.5
1.0
0.5
0.0
Ctrl
Cd
PQ
Cd+PQ
g
Ctrl
Cd
PQ
Cd+PQ
Counts
MitoTracker Red
Fluorescence Intensity
h
Ctrl
Cd
PQ
Cd+PQ
Upper
2.98
Upper
3.93
Upper
3.29
Upper
6.20
pH4 (561nm)
pH7 (488nm)

Fig. S6

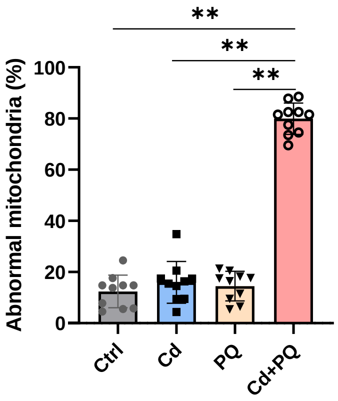

Supplement: Supplementary file 1 — Supplementary figures. [file ijbsv22p2885s1.pdf]
